# Effects of Kuanxiong aerosol for preventing acute mountain sickness: an exploratory small-sample randomized, placebo-controlled trial

**DOI:** 10.3389/fphys.2026.1834301

**Published:** 2026-07-21

**Authors:** Yi-xiu Lin, Peng-fei Zhang, Zhe-tao Wang, Dan Li, Jiao Luo, Shu-ming Ji, Cheng Huang, Lei Chen

**Affiliations:** 1 Department of High Altitude Medicine, Center for High Altitude Medicine, West China Hospital, Sichuan University, Chengdu, China; 2 Department of Neurology, West China Hospital, Sichuan University, Chengdu, Sichuan, China; 3 Department of Radiology, West China Hospital, Sichuan University, Chengdu, Sichuan, China; 4 Department of Rehabilitation Medicine, Center for High Altitude Medicine, West China Hospital, Sichuan University, Chengdu, Sichuan, China; 5 Key Laboratory of Rehabilitation Medicine in Sichuan Province, West China Hospital, Sichuan University, Chengdu, Sichuan, China; 6 West China School of Nursing, Sichuan University, Chengdu, Sichuan, China; 7 Department of Clinical Research Management, West China Hospital, Sichuan University, Chengdu, China

**Keywords:** acute mountain sickness, high altitude, hypobaric hypoxia, Kuanxiong aerosol, prevention

## Abstract

**Background:**

This study aimed to evaluate the potential efficacy and neurophysiological effects of Kuanxiong Aerosol (KXA) in preventing acute mountain sickness (AMS) during simulated high-altitude exposure.

**Methods:**

In this randomized, double-blind, placebo-controlled trial, 20 healthy volunteers underwent a 6-hour hypobaric hypoxia session (simulated 4000 m) after receiving either KXA or placebo. Outcomes included the Lake Louise Score (LLS), AMS incidence, physiological parameters (SpO_2_, heart rate), cognitive performance (PMT, Symbol-Digit Modalities Test), and cerebral blood flow (CBF) measured by MRI. An exploratory *post-hoc* subgroup analysis was also conducted in nine participants who voluntarily received an additional KXA session in a self-controlled manner.

**Results:**

In the main trial, KXA administration was associated with a non-significant reduction in total LLS and a decrease in AMS incidence from 50% (placebo) to 30% (KXA). No significant differences were observed in SpO_2_, cognitive tests, or global CBF. However, the KXA group showed a smaller increase and more stable heart rate profile over time. Exploratory regional CBF analyses revealed reduced perfusion in selected subregions of the limbic and control networks in the KXA group (*p* < 0.05 uncorrected). In the post-hoc self-controlled subgroup (n = 9), KXA significantly reduced total LLS (*p* = 0.037) and decreased AMS incidence from 44% to 0%, with trends toward improved cognitive performance.

**Conclusion:**

KXA shows preliminary potential for alleviating AMS symptoms under simulated high-altitude conditions, possibly through cardiac and regional cerebrovascular modulation. These findings support further investigation in larger, randomized controlled trials in real-world high-altitude settings to confirm efficacy and clarify the underlying mechanisms.

**Chinese clinical trial registry:**

ChiCTR2500105469.

## 1 Introduction

High-altitude regions are generally defined as areas above 2,500 meters. Characterized by low atmospheric pressure, hypoxia, strong ultraviolet radiation, low temperatures, and large diurnal temperature variations, these environments can readily impair human physiological function and contribute to altitude-related disorders. In China, which possesses extensive plateau terrain and a large high-altitude population, the prevention and treatment of such diseases constitute a significant public health priority. Acute altitude sickness encompasses a range of pathophysiological responses triggered by insufficient adaptation following rapid ascent, primarily including acute mountain sickness (AMS), high-altitude pulmonary edema (HAPE), and high-altitude cerebral edema (HACE) (Luks et al., 2024). AMS is the most common form, typically manifesting within 4–12 hours of arrival with symptoms such as headache, dizziness, nausea, and fatigue (Simancas-Racines et al., 2018).

The incidence of AMS is closely linked to the rate of ascent, final altitude, and individual susceptibility. Studies indicate that approximately 25% of individuals develop AMS at moderate altitudes, with incidence rising to 50–85% among travelers above 4,000 meters (Eide and Asplund, 2012). Females and individuals with a history of migraine are at elevated risk (Canoui-Poitrine et al., 2014). The pathogenesis of AMS is multifactorial and not fully understood, though acute hypoxia is considered the primary trigger (West and Richalet, 2013). Systemically, acute hypoxia initiates adaptive responses, including hyperventilation mediated by the hypoxic ventilatory response (HVR) (Weil et al., 1970) and increased heart rate and cardiac output driven by sympathetic activation (Bartsch and Gibbs, 2007), both aimed at enhancing tissue oxygen delivery.

Current AMS prevention primarily relies on gradual acclimatization and supplemental oxygen. Commonly used agents include: Acetazolamide, dexamethasone, and ibuprofen, but each has limitations and side effects (Gertsch et al., 2012; Basaran et al., 2016; Tapia and Irarrazaval, 2019). Therefore, new preventive strategies are needed. Traditional Chinese medicine has accumulated substantial experience in preventing and treating high-altitude disorders. Commonly used formulations include Compound Danshen Dripping Pills, Shexiang Baoxin Pills, Naokang Pills, and Rhodiola Capsules (Yan et al., 2010). Their mechanisms are thought to involve reducing oxygen consumption, enhancing hypoxia tolerance, and mitigating hypoxic injury (Yi et al., 2015). They may also elevate superoxide dismutase (SOD) activity, suppress inflammation, and modulate autophagy (HJ et al., 2019).

Preliminary studies suggest that KXA can improve myocardial ischemia, increase coronary blood flow, ameliorate microvascular dysfunction (Huang et al., 2022; Liu et al., 2025). Its sublingual administration allows rapid enhancement of myocardial oxygen supply and relief of chest tightness. Cellular and animal experiments indicate that KXA alleviates myocardial hypoxia, inhibits apoptosis, and can cross the blood–brain barrier (BBB) (Sun et al., 2025; Chen et al., 2026). Using an *in vitro* Transwell model and an *in vivo* t-MCAO mouse model, Chen et al. (2026) demonstrated that components of KXA can cross the BBB, with GC-MS analysis confirming the presence of eucalyptol, and isoborneol in brain tissue.

In models of high-altitude-related cerebral symptoms, KXA demonstrated neuroprotective effects in cerebral ischemia–reperfusion (t-MCAO) models, reducing infarct volume and improving neurological deficits and motor coordination. Further *in vitro* studies confirm that KXA inhibits TRPV1 channel activity, reduces calcium influx, restores mitochondrial membrane potential, and alleviates oxidative stress in neuronal cells, thereby mitigating calcium overload and protecting mitochondrial function (Chen et al., 2026).

Building on prior research on KXA and practical feedback from high-altitude regions, this study aims to evaluate its efficacy and safety in preventing and treating acute high-altitude sickness, with the goal of providing new clinical strategies for its management.

## 2 Methods

### 2.1 Study design and settings

This study was a single-center, randomized, parallel-group, double-blind, prospective clinical trial, enrolling healthy subjects from lowland areas who plan to travel to high-altitude regions. Participants were randomly assigned to either the KXA group or the placebo group and entered the hypobaric hypoxic chamber (12% oxygen, 88% nitrogen, simulating an altitude of approximately 4,000 meters). A placebo aerosol that was identical in appearance, packaging, and administration procedure to the KXA was used in the placebo group. The dosing regimen began one day prior to entry (KXA or placebo): three puffs per dose, three times daily. On the day of entry, one dose (three puffs) was administered in the morning before entering the chamber, and two additional doses (three puffs each one) were administered after chamber entry, with a 4–5 hours interval between the two doses.

The efficacy and safety of KXA in the prevention of acute mountain sickness were evaluated by assessing the incidence and severity of symptoms, monitoring physiological parameters, and recording adverse effects.

The hypobaric chamber was maintained at a steady-state temperature of 18–24 °C and relative humidity of 40–60% RH throughout the experiment. These environmental parameters were continuously monitored and kept consistent across the control and KXA sessions to minimize any potential confounding effects on physiological responses.

### 2.2 Participants

Our inclusion criteria encompass: healthy volunteers planning travel or work at high altitudes, regardless of gender, aged 18–55 years (including boundary values); primary residence at an altitude ≤800 meters; no elevation exceeding 2500 meters within 6 months prior to screening; and subjects who voluntarily participate and provide written informed consent.

Exclusion criteria include: severe high-altitude reactions within the past 5 years, including high-altitude cardiac syndrome, high-altitude pulmonary edema, high-altitude cerebral edema, and high-altitude polycythemia; previously diagnosed cardiovascular or cerebrovascular disease, or uncontrolled hypertension (screening systolic blood pressure ≥140 mmHg and/or diastolic blood pressure ≥90 mmHg);

Clinically significant respiratory, digestive, hepatic, central nervous system, psychiatric, metabolic, renal diseases, anemia, or acute infections; psychiatric disorders (including anxiety, depression, and insomnia) and migraine.

Primary headaches (migraine, tension headache, cluster headache, etc.) or secondary headaches (headaches related to infection, cerebrovascular disease, etc.) within one month prior to screening; LLS score ≥1 at screening; left index finger oxygen saturation <95% at screening; ALT or AST > 2 times the upper limit of normal range at screening, or creatinine > upper limit of normal range; use of any medication or non-pharmacological intervention (including dietary supplements) within 14 days prior to screening for prevention or treatment of acute mountain sickness; history of cardiopulmonary disease, respiratory infection within 3 months; Contraindications to MRI scanning, or prior MRI evidence of brain disease; Individuals with contraindications or allergies to medications used in the study; Breastfeeding, or women with positive pregnancy tests; Participation in other interventional clinical studies within 3 months prior to screening; Daily routine use of one or more medications; Habitual smoking or heavy alcohol consumption; Alcohol or coffee consumption within 3 days prior to screening.

### 2.3 Outcomes

Primary outcome measures included the incidence of AMS and LLS between the two groups. Secondary outcome measures included headache visual analog scale (VAS) scores, processing speed scale scores, changes in blood oxygen saturation, blood pressure, and heart rate during simulated hypoxia in a hypobaric chamber, executive function scale scores, memory scale scores, and magnetic resonance imaging (MRI).

### 2.4 Sampling collection and analyses

This study employed a randomization method based on drawing numbered balls. Twenty-four balls, identical in shape and size and numbered from 01 to 24, were prepared and thoroughly mixed. Subjects who drew an odd number were assigned to the KXA group, while those who drew an even number were assigned to the placebo group. To ensure double-blind conditions, the KXA and the placebo were identical in appearance, packaging, and labeling.

Baseline data were collected from the subjects prior to their entry into the low-pressure oxygen chamber. This included clinical information, heart rate (HR), peripheral oxygen saturation (SpO_2_), arterial spin labeling (ASL) brain magnetic resonance imaging (MRI), as well as assessments using the Picture Memory test (PMT) for memory, the Symbol Digit Modalities Test (SDMT), and the LLS. PMT presented pictures sequentially at 1-second intervals; after the sequence, participants clicked the pictures in the original order. The SDMT required participants to select the symbol corresponding to a given digit. Both tests were provided by the Beijing Jingshi Brain Research Institute.

**Figure 1:**
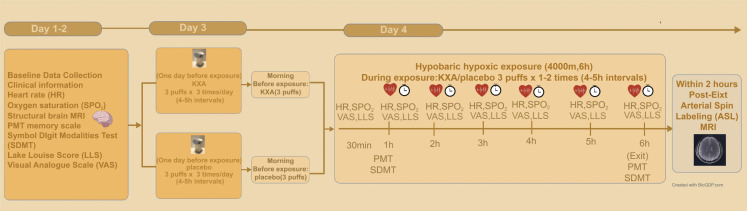
Study flowchart: Participants were exposed to a simulated altitude of 4000 m for 6 hours in a hypobaric chamber. Physiological monitoring (SpO_2_, HR) and behavioral assessments (LLS, VAS, PMT, SDMT) were performed at specific intervals. ASL-MRI scans were acquired at baseline and within 2 hours after exiting the chamber.

They were randomly assigned to either the KXA group or the placebo group. The dosing regimen was as follows: starting one day before exposure, 3 sprays per dose, three times a day, with 4–5 hours between doses. On the day of exposure, one dose (3 sprays) was administered in the morning before entering the chamber. After entering the chamber, 2 additional doses (3 sprays each) were administered as indicated, with an interval of 4–5 hours. Subsequently, the subjects entered a chamber simulating the low-pressure and low-oxygen conditions at an altitude of 4000 meters for a 6-hour exposure. HR, SpO_2_, and headache severity using a VAS were monitored at 0.5, 1, 2, 3, 4, 5, and 6 hours after entering the chamber. The LLS was also assessed at these same time points. PMT and SDMT evaluations were conducted at the 1-hour and 6-hour time points. An ASL-MRI scan was completed within 2 hours after exiting the chamber [**Figure 1**; Created with BioGDP (Jiang et al., 2025)].

### 2.5 Exploratory voluntary additional session

After completion of the main trial and after unblinding, participants from the placebo group who were planning imminent high-altitude work were offered the opportunity to receive hypoxic preconditioning plus KXA as a potential personal preventive measure within one week before their actual high-altitude deployment. Nine of the 10 placebo-assigned participants voluntarily chose to re-enter the chamber 14 days after their first session.

The session followed the same protocol as the main trial: 6-h exposure to simulated 4000 m altitude (12% O_2_, 88% N_2_), with KXA administered according to the same dosing regimen (3 puffs three times daily starting one day before, plus one dose before entry and two doses after entry, 4-5 h apart). Physiological monitoring (SpO_2_, HR, VAS, LLS), cognitive tests (PMT, SDMT), and ASL-MRI were performed at the same time points as in the main trial. However, two of the nine participants declined the MRI scan; therefore, ASL-MRI data are available for only seven participants.

This additional session was not part of the registered protocol. Participants gave separate written informed consent. Because there was no placebo control and the design was open-label, the analysis is exploratory and hypothesis-generating.

### 2.6 MRI data acquisition

MRI data were acquired using a GE 3.0T scanner (GE Healthcare, premier, Milwaukee, WI, USA). High-resolution 3D T1-weighted images were acquired (3D-BRAVO): TR/TE = 6.2/2.5 ms,

FOV=256 × 256mm2, voxel size= 1×1×1mm2. Whole-brain perfusion was assessed using a 3D pCASL sequence: TR = 4832 ms, TE = 51.1 ms, post-labeling delay (PLD) = 2025 ms, flip angle = 17°, FOV = 229×229 mm², acquisition voxel size = 0.7×0.7×4.0 mm³, slices = 52 (Alsop et al., 2015). Quantitative CBF maps were reconstructed on the scanner.

### 2.7 MRI data processing and metric extraction

MRI data were processed using SPM12. Individual CBF maps were first co-registered to the corresponding T1-weighted images. The T1-weighted images were then segmented and normalized to the Montreal Neurological Institute (MNI) space using the DARTEL algorithm implemented in SPM12. The resulting deformation fields were subsequently applied to the co-registered CBF maps, which were resampled to 3 × 3 × 3 mm³ in MNI space. No spatial smoothing was applied to the normalized CBF maps in order to minimize signal blurring and preserve regional specificity. Gray matter (GM) and white matter (WM) probability maps derived from T1 segmentation were thresholded at > 0.2 to generate tissue masks, from which mean CBF values of the whole brain, GM, and WM were extracted. For ROI-level analysis, the Schaefer atlas (400 parcels, 7 networks) was overlaid onto the normalized unsmoothed CBF maps in MNI space to extract the mean CBF value for each ROI (Yeo et al., 2011; Schaefer et al., 2018).

### 2.8 Statistical analyses

Statistical analyses were performed using SPSS (version 27.0), Free Statistics V2.2.0 (Ruan et al., 2022) and MATLAB. Normality was assessed with the Shapiro-Wilk test. Data are presented as mean ± standard deviation (SD) or median [interquartile range, IQR]. Categorical variables are expressed as frequencies and percentages. The incidence of AMS between sessions was compared using the Chi-square test. For longitudinal measurements (SpO_2_, HR, VAS, PMT, SDMT), a Repeated Measures Analysis of Variance (ANOVA) was applied to evaluate changes across time points and their interaction with the intervention.

A one-way repeated-measures ANOVA was conducted to evaluate CBF changes across the three MRI time points (Baseline, Post-Control, and Post-Intervention). For ROI-level analyses, p-values from the ANOVA were corrected for multiple comparisons across ROIs using the false discovery rate (FDR) method. As no ROI survived ROI-level multiple-comparison correction, all ROI-level ANOVA statistics (including *F* and *p* values) are reported in detail for transparency. Exploratory *post-hoc* paired *t*-tests were further performed to compare CBF between sessions (Baseline vs. Post-Control, Baseline vs. Post-Intervention, and Post-Control vs. Post-Intervention). To account for the three pairwise session comparisons, a Bonferroni-adjusted significance threshold of *p* < 0.05/3 was applied. These *post-hoc* analyses were not further corrected for the number of ROIs and should therefore be interpreted as exploratory.

### 2.9 Study approval

The study was approved by the Ethics Committee of the West China Hospital, Sichuan University and registered on Chinese Clinical Trial Registry (ChiCTR2500105469). All participants provided written informed consent. The study was conducted in compliance with the Declaration of Helsinki.

## 3 Results

### 3.1 Participant characteristics

**Figure 2:**
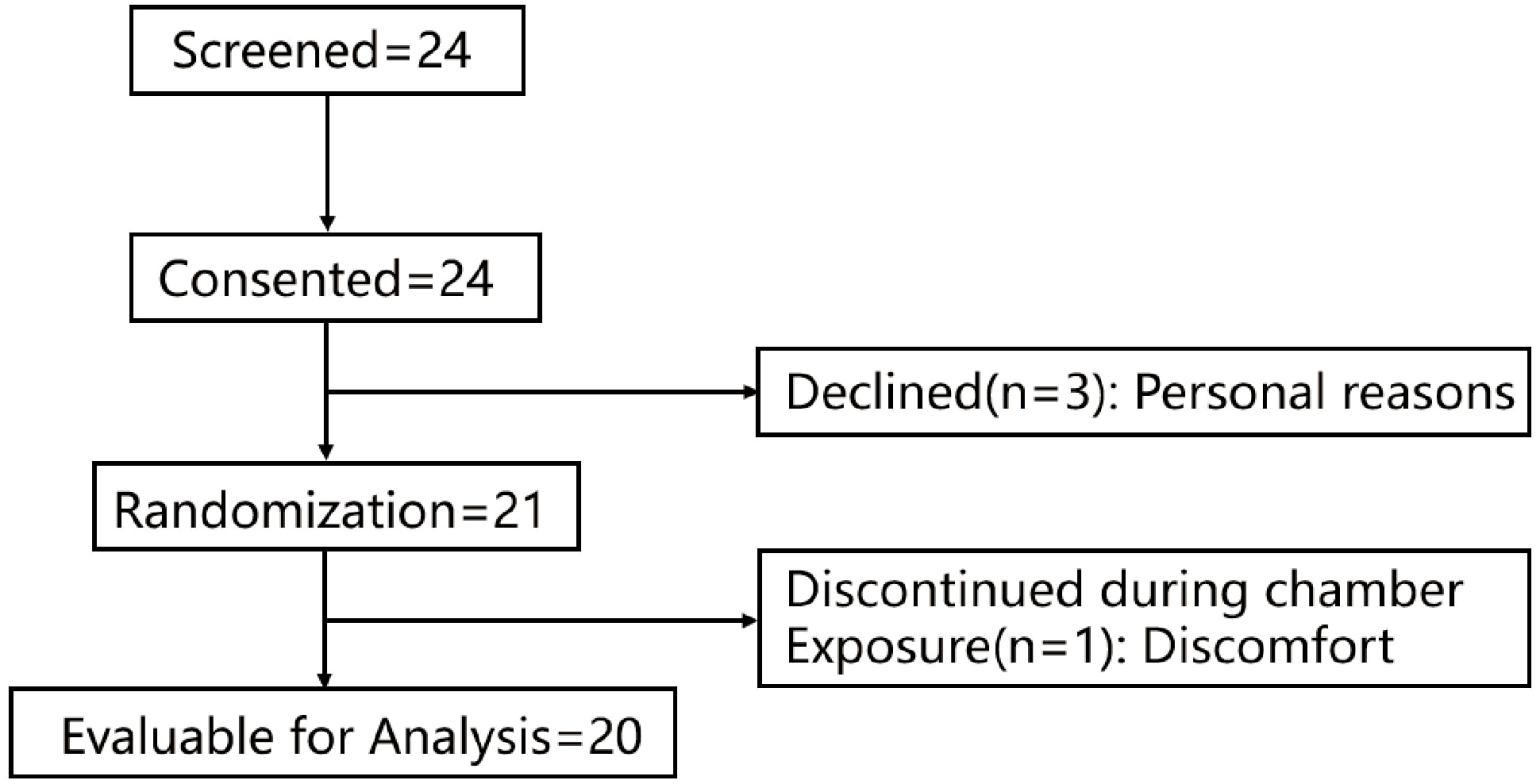
Participant flow study diagram: describes participant flow through the study.

Of 24 participants who gave informed consent, three withdrew before randomization for personal reasons, and one discontinued during chamber exposure due to discomfort. A total of 20 participants were included in this study (**Figure 2**). **Table 1** summarized the demographic and clinical characteristics of all participants. No significant baseline differences were observed between the KXA and placebo groups.

**Table 1:** Baseline demographic and clinical characteristics of the study participants.

Variables	Total (n=20)	KXA group (n=10)	Control group (n=10)	P
Sex, n (%)				0.65
Female	12 (60)	5 (50)	7 (70)	
Male	8 (40)	5 (50)	3 (30)	
Age, Mean ± SD	26.6 ± 6.2	24.7± 3.3	28.5± 7.9	0.178
Past medical history (comorbidities), n (%)				1
Absent	20 (100.0)	10 (100)	10(100)	
Surgical history, n (%)				0.087
Absent	16 (80)	10(100)	6(60)	
Present	4(20)	0 (0)	4(40)	
History of headache (within the past month), n (%)				1
Absent	20 (100.0)	10 (100)	10 (100)	
History of high-altitude travel, n (%)				0.35
Absent	7 (35)	2 (20)	5 (50)	
Present	13 (65)	8 (80)	5 (50)	
History of altitude sickness, n (%)				0.35
Absent	13 (65)	5 (50)	8 (80)	
Present	7 (35)	5 (50)	2 (20)	
Smoking status, n (%)				1
Absent	20 (100.0)	10 (100)	10 (100)	
Alcohol use, n (%)				0.087
Absent	16 (80)	6 (60)	10 (100)	
Present	4 (20)	4 (40)	0 (0)	
BMI, Mean	21.8 ± 3.5	21.6± 3.5	22.0± 3.6	0.806
Lake Louise Scale scores (LLS)	0 ± 0	0 ± 0	0 ± 0	1

Data are presented as mean ± standard deviation (SD) for continuous variables or number (percentage) for categorical variables. All participants reported a Lake Louise Score (LLS) of 0 at baseline.

### 3.2 Primary outcomes

The use of KXA led to a mitigation of acute mountain sickness symptoms in the simulated high-altitude environment, although the differences did not reach statistical significance. The primary outcome, the total Lake Louise Score, was reduced following drug intervention, from (3.0 (1.2, 4.8) in the placebo group to 1.5 (1.0, 2.8) in the KXA group (p = 0.467; **Table 2**). Furthermore, a non-significant trend was observed in the incidence of AMS (LLS ≥ 3 with headache) (Roach et al., 2018), which decreased from 50% (5/10) with placebo to 30% with KXA (*p* = 0.65; **Table 2**). Other individual symptom scores, including headache and fatigue, were also lower with KXA (**Table 2**). None of these reductions achieved statistical significance.

**Table 2:** Comparison of Lake Louise Scale scores between the KXA group and the placebo group under simulated high-altitude exposure.

Variables	Total(n=20)	KXA group (n=10)	Control group (n=10)	P
Total LLS Score (IQR)	2.0 (1.0, 4.2)	1.5 (1.0, 2.8)	3.0 (1.2, 4.8)	0.467
Acute Mountain Sickness (AMS), n (%)				0.65
Absent (LLS < 3 or no headache)	12(60)	7(70)	5 (50)	
Present (LLS ≥ 3 and headache)	8(40)	3(30)	5(50)	
Headache, Mean ± SD	0.6± 0.5	0.5 ± 0.5	0.6 ± 0.5	0.673
Gastrointestinal symptoms, Mean ± SD	0.3 ± 0.5	0.3 ± 0.5	0.3 ± 0.5	1
Fatigue/weakness, Mean ± SD	0.5 ± 0.5	0.4 ± 0.5	0.6 ± 0.5	0.398
Dizziness, Mean ± SD	0.4 ± 0.5	0.5 ± 0.5	0.3 ± 0.5	0.388

KXA, Kuanxiong Aerosol; LLS, Lake Louise Score; AMS, Acute Mountain Sickness; SD, standard deviation. Data Calculation: The “Total LLS Score” and individual symptom scores represent the maximum value (peak score) recorded for each participant across all monitoring time points during the 6-hour hypoxic exposure. Definitions: AMS was diagnosed if the peak Total LLS was ≥ 3 with the presence of a headache.

Data are presented as median (IQR) for total LLS score and as mean ± SD for individual symptom scores. Comparisons between the KXA and placebo groups were performed using the Mann-Whitney U test for total LLS score, the independent samples t-test for individual symptom scores, and Fisher’s exact test for AMS incidence.

### 3.3 Secondary outcomes

**Figure 3:**
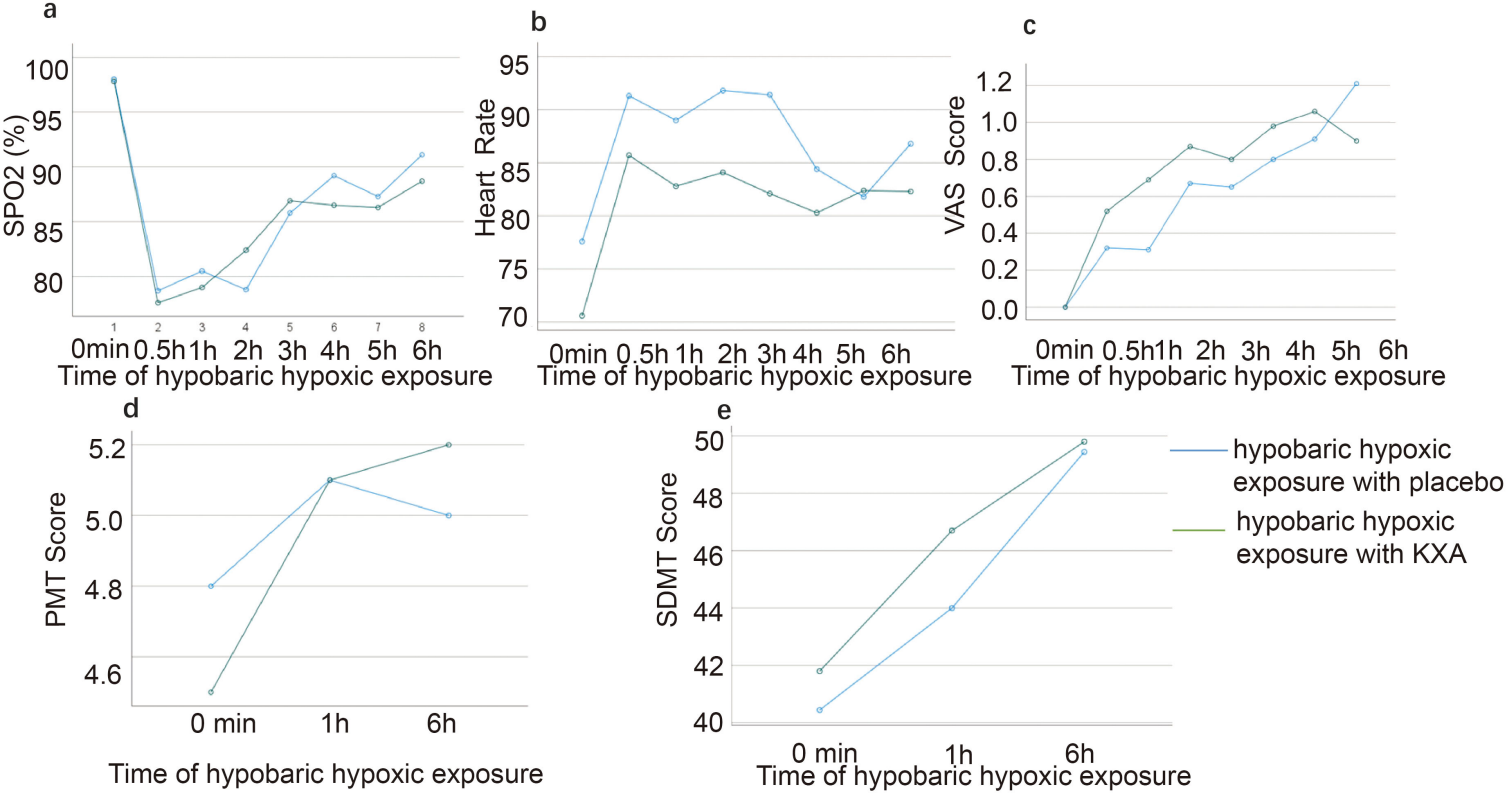
Temporal trends in physiological and cognitive outcomes during the KXA and placebo group. **(a)**. Peripheral oxygen saturation (SpO_2_). **(b)**. heart rate (HR), and **(c)**. headache severity (VAS) assessed hourly. Picture Memory Test [PMT, **(d)**] and Symbol-Digit Modalities Test **(e)** scores at baseline, 1h, and 6h. The blue line indicates hypobaric exposure with placebo, and the green line indicates hypobaric exposure with KXA.

SpO_2_, heart rate (HR), blood pressure (BP), and headache visual analogue scale (VAS) scores were measured at baseline and at 1-hour intervals throughout the low-pressure hypoxic exposure. The PMT memory test and the Symbol-Digit Modalities Test (SDMT) were administered at baseline, 3 h, and 6 h of exposure. Of note, one participant in the control group refused to complete the SDMT; therefore, the final number of participants included for SDMT analysis was 9 in the control group. significant main effect of time was observed for SpO_2_, HR, BP, VAS, and both cognitive tests (all *P* < 0.05). However, no statistically significant differences were detected between the two exposure sessions (with versus without KXA) for any of these outcomes (all *p* > 0.30). Although no statistical significance was reached, consistent directional trends were observed: compared with placebo, KXA tended to lower VAS scores and increase scores on both the PMT and SDMT (**Figure 3**; **Supplementary Figure 1**).

### 3.4 Exploratory neuroimaging findings

**Figure 4:**
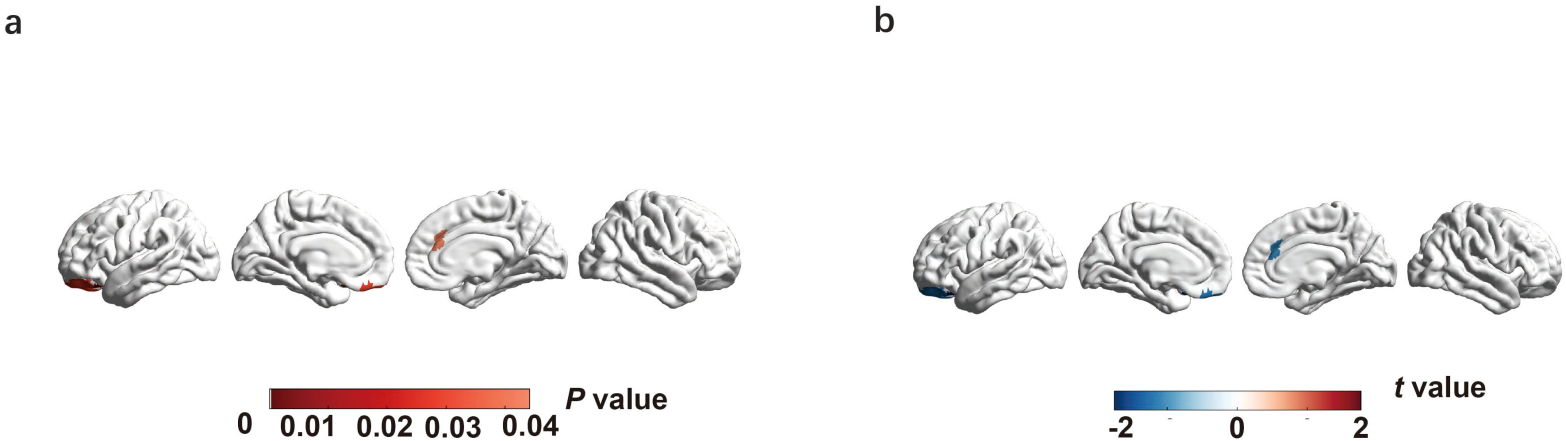
Differences in regional cerebral blood flow (CBF) between the placebo group and the KXA group. **(a)**. P-values for the comparison of regional CBF between the two groups (uncorrected). **(b)**. Corresponding t-values for the comparison of regional CBF between the two groups (uncorrected). Only regions with p < 0.05 (uncorrected) are displayed. None of these differences survived FDR correction for multiple comparisons. ROI analyses were performed using the Schaefer 400-parcel atlas (7-network version). Cool colors represent decreased CBF, and warm colors represent increased CBF. The significance threshold was set at *p* < 0.05 (uncorrected). CBF, cerebral blood flow; ROI, region of interest; FDR, false discovery rate.

Exploratory ROI-based analyses were performed to compare regional CBF between the KXA and placebo groups after hypobaric hypoxic exposure. No significant differences were observed in global or gray matter CBF. However, at the regional level, the KXA group showed significantly lower CBF in three ROIs compared with the placebo group (*p* < 0.05, uncorrected): the left orbitofrontal cortex (two clusters, belonging to the limbic network; ROI identifiers 114 and 116) and the right prefrontal cingulate cortex (control network; ROI 360) (**Figure 4**; **Supplementary Table 1**). After controlling for multiple comparisons using the false discovery rate (FDR) method, none of these differences remained significant (q > 0.05). Therefore, these regional findings are considered exploratory and hypothesis-generating (**Supplementary Table 2**).

### 3.5 Exploratory voluntary additional session

In an exploratory analysis of nine participants who initially received placebo in the main trial and later voluntarily received KXA (self-controlled comparison), KXA administration was associated with a significant reduction in total Lake Louise Score compared with their own placebo session (p = 0.037; **Table 3**). Headache severity also improved (**Table 3**). The incidence of AMS decreased from 44% (4/9) during the placebo session to 0% (0/9) after KXA, although this difference did not reach statistical significance (**Table 3**). Cognitive performance (PMT, SDMT) showed increasing trends, while VAS scores tended to decrease (**Supplementary Figure 1**). Neuroimaging revealed a distinct neurovascular response, characterized by reduced global cerebral blood flow (CBF) and a redistribution of perfusion favoring higher order cognitive networks over somatomotor and salience/ventral attention networks (**Supplementary Figure 2**; **Supplementary Figure 3**; **Supplementary Table 3**; **Supplementary Table 4**).

**Table 3:** Comparison of Lake Louise Scale scores between the placebo session and KXA session under simulated high-altitude exposure (self-controlled, N = 9).

Variables	First exposure (with placebo) (n=9)	Second exposure (with KXA aerosol) (n=9)	p
Total LLS Score, Mean ± SD	2.8 ± 2.3	0.9 ± 0.6	0.037
Acute Mountain Sickness (AMS), n (%)			0.063
Absent (LLS < 3 or no headache)	5 (55.6)	9 (100)	
Present (LLS ≥ 3 and headache)	4 (44.4)	0 (0)	
Headache, Mean ± SD	0.6 ± 0.5	0.1 ± 0.3	0.035
Gastrointestinal symptoms, Mean ± SD	0.3 ± 0.5	0.0 ± 0.0	0.081
Fatigue/weakness, Mean ± SD	0.7 ± 0.5	0.6 ± 0.5	0.681
Dizziness, Mean ± SD	0.3 ± 0.5	0.1 ± 0.3	0.347

KXA, Kuanxiong Aerosol; LLS, Lake Louise Score; AMS, Acute Mountain Sickness; SD, standard deviation. Data Calculation: The “Total LLS Score” and individual symptom scores represent the maximum value (peak score) recorded for each participant across all monitoring time points during the 6-hour hypoxic exposure. Definitions: AMS was diagnosed if the peak Total LLS was ≥ 3 with the presence of a headache.

Data are presented as mean ± SD or frequency (%). *P*-values indicate differences between the First Exposure (Control) and Second Exposure (KXA Intervention). Comparisons were performed using the paired samples ***t***-test for continuous scores and McNemar’s test for AMS incidence.

## 4 Discussion

This exploratory, small-sample, randomized controlled trial provided preliminary evidence that KXA may alleviate acute mountain sickness (AMS) during simulated high-altitude exposure. Compared with the control group, KXA administration was associated with a reduction in the Lake Louise Scale (LLS) total score and a trend toward a lower incidence of AMS (decreasing from 50% to 30%). No significant differences in oxygen saturation were observed between the two groups; however, relative to the control group, the KXA group showed a smaller increase in heart rate (HR) and a more stable HR profile over time. Supportive directional trends were also observed in subjective headache severity and objective cognitive performance. None of these results reached statistical significance in this small sample. In the MRI analysis, compared with the placebo group, the KXA group showed reduced perfusion in some subregions of the limbic network and the control network; however, these differences did not survive FDR correction for multiple comparisons. In summary, these preliminary findings suggest that KXA may alleviate symptoms of acute mountain sickness, may reduce cardiac workload, and might support cognitive function during acute hypoxic stress, although confirmatory trials are needed.

Furthermore, in an exploratory post-hoc subgroup analysis of nine participants who initially received placebo and later voluntarily received KXA (self-controlled), total LLS scores were significantly lower after KXA than after placebo (*p* = 0.037), and AMS incidence decreased from 44% to 0%. These findings are consistent with the trends observed in the main study and support the possibility that KXA may have a preventive effect on AMS. However, the significant reduction in LLS scores in this exploratory subgroup might also be related to the hypoxic exposure experienced during the first chamber entry. Studies have shown that hypoxic preconditioning may prevent AMS (You et al., 2023).

In our study, 6 h after rapid ascent to high altitude, the mean blood oxygen saturation levels in both the KXA group and the placebo group ranged from 87% to 92%, which is similar to the 80%–90% range reported in previous studies conducted at altitudes of 3,000–4,000 m (Luks and Swenson, 2011). However, unlike currently used medications for the prevention of acute mountain sickness that can increase SpO_2_ following acute hypoxia, no significant increase in SpO_2_ was observed in the KXA group, a finding that may be related to our small sample size (Liu et al., 2024). Furthermore, in high-altitude hypoxic environments, heart rate increases to accelerate blood circulation and ensure oxygen supply to vital organs (Naeije, 2010). The overall increase in heart rate was smaller in the KXA group, which may be related to bronchodilation and reduced cardiac workload (Qian et al., 2026). We speculate that the preventive effect of KXA against acute mountain sickness may be achieved through cardiac regulation.

Acute high-altitude exposure causes multiple cognitive impairments, affecting people’s work and daily life at high altitudes (Taylor et al., 2015). The Symbol Digit Modalities Test (SDMT) evaluates processing speed and accuracy by requiring subjects to match symbols with corresponding digits within a time limit. This task engages visual perception, working memory, attentional control, and visuomotor coordination, serving as a well-established measure of attention and executive function (Flaim and Blaisdell, 2020; Rubaiyat et al., 2024). Previous studies have found that in high-altitude environments, sustained attention duration shortens and attention switching (AST) task scores decline (De Bels et al., 2019). In the PMT, a series of objects are presented in a fixed order on a computer screen. Participants must memorize and reproduce these items during a learning trial, a process that involves the acquisition, storage, and recall of new information. Previous studies have also confirmed that high-altitude exposure impairs memory function, particularly short-term memory (Chen et al., 2023). In this small exploratory sample, between-session comparisons of test scores did not reach statistical significance. Nevertheless, the KXA-treated group tended to have higher PMT and SDMT scores than the placebo group, suggesting that KXA may exert a beneficial modulatory effect on cognitive domains particularly susceptible to hypoxia, especially executive attention and episodic memory encoding/retrieval. In an exploratory post-hoc analysis of the nine participants who received KXA in a voluntary additional session (self-controlled comparison), SDMT and PMT scores were also higher during the KXA session than during their first exposure when placebo was administered. Although these findings are hypothesis-generating and lack a control group, they directionally support the primary analysis.

In high altitude, alterations in cerebral blood flow and functional connectivity can also be observed. Villien et al. found that exposure to high altitude significantly increased blood flow during the stay at altitude and within 6 hours after returning to sea level (Villien et al., 2013). Under high-altitude exposure, blood oxygen content decreases significantly. To maintain cerebral oxygen supply, cerebral arteries dilate, with marked increases in the cross-sectional area of the carotid, basilar, and midbrain arteries, thereby rapidly enhancing cerebral blood flow (Ainslie and Subudhi, 2014; Liu et al., 2017). Acute high-altitude exposure leads to a significant reduction in global brain efficiency and impaired functional connectivity (Liu et al., 2022). Multiple brain regions involved in attention control, response, memory, and motor coordination exhibit changes in centrality and node efficiency (Xin et al., 2020; Lu et al., 2022).Alterations in brain network activation levels are also observed during cognitive tasks (Li et al., 2020; Lu et al., 2022). In our study, we observed changes in cerebral blood flow perfusion in the KXA group compared with the placebo group after exposure to a simulated high-altitude environment. Specifically, three brain regions in the KXA group—the left orbitofrontal cortex (two subregions of the limbic network) and the right prefrontal cingulate cortex (part of the control network)—showed lower perfusion than those in the placebo group (p < 0.05, uncorrected). These regions are associated with emotion and cognitive control. Furthermore, exploratory subgroup analyses also revealed that KXA administration reduced cerebral blood flow relative to placebo and redistributed perfusion to prioritize higher-order cognitive networks. These findings may suggest that KXA modulates perfusion in specific brain regions; however, these differences did not survive FDR correction and are therefore inconsistent, requiring verification in a larger sample.

Based on the above findings and in light of the existing literature, we hypothesized that KXA might exert its effects in acute high-altitude sickness through the following mechanisms: (1) Certain components of KXA could cross the blood-brain barrier (Chen et al., 2026), downregulate TRPV1 channel expression, inhibit calcium influx, and alleviate oxidative stress (Chen et al., 2026). TRPV1 channels are known to be involved in the regulation of cerebral vascular tone (North et al., 2018; Negri et al., 2020). Under hypoxic conditions, KXA might modulate the activity of these channels, thereby influencing microvascular tone or the efficiency of neurovascular coupling. (2) KXA might improve cardiac function and hemodynamic stability (Morrell et al., 2007). (3) By inhibiting calcium influx and activating the CaMKII/ERK pathway, KXA might induce vasodilation, improve blood rheology, and optimize oxygen delivery efficiency (Lu et al., 2022). (4) KXA might also attenuate hypoxic inflammatory responses via the cGAS-STING and NOTCH1 signaling pathways (Mack et al., 2017; Lu et al., 2022; Chen et al., 2025; Sun et al., 2025), thereby indirectly protecting neurovascular integrity. These hypotheses require further investigation using pharmacokinetic data and central-specific biomarkers.

Several limitations should be acknowledged. First, the small sample size (20 participants in the main RCT) limited statistical power and may have contributed to the failure to detect significant differences in the primary clinical outcomes, as well as the lack of significant global CBF changes. Second, the neuroimaging findings at the regional level did not survive correction for multiple comparisons and should therefore be considered exploratory and hypothesis−generating. Third, the controlled hypobaric hypoxic chamber setting differs from real−world high−altitude conditions in terms of physical activity, exposure duration, and psychological stress, necessitating validation in field−based trials with larger cohorts. Fourth, the voluntary additional session (n=9) lacked a control group and was not part of the registered protocol; its results are only supportive. Fifth, no pharmacokinetic (PK) data are available to support the chosen dosing regimen; In addition, there were some limitations in MRI acquisition due to methodological and technical constraints. The single post-labeling delay (PLD) of 2025 ms was suitable for normoxic conditions but may not be fully applicable to the prolonged arterial transit time under hypobaric hypoxia caused by cerebral vasoconstriction. Moreover, MRI was performed within 2 hours after hypoxic exposure, rather than during or immediately after hypoxia. Upon re-exposure to a normoxic environment, CBF begins to recover toward baseline levels; therefore, partial recovery may have occurred before scanning. Last, our stringent exclusion criteria (e.g., LLS ≥ 1 at screening, age 18–55, SpO_2_ < 95%, ALT/AST > 2× ULN, and exclusion of headache-prone individuals) were adopted to maximize internal validity in this pilot exploratory study, but they limit generalizability to real-world high-altitude travelers. We therefore recommend that future larger trials adopt more inclusive criteria: reduce primary headache exclusion to 7 days, use a higher LLS threshold or only exclude specific AMS-like symptoms, shorten respiratory infection exclusion to 14 days, and restrict coffee/alcohol intake to 24-48 h with clear definitions of habitual use. For headache-prone individuals, we suggest stratification by headache history using validated tools (e.g., ID-Migraine) rather than outright exclusion.

## 5 Conclusion

In this small−sample, exploratory randomized controlled trial, KXA aerosol showed non−significant directional improvements in AMS symptoms, heart rate stability, and cognitive performance compared with placebo. Neuroimaging analysis revealed exploratory regional perfusion changes in the limbic and control networks, although these findings did not withstand correction for multiple comparisons. An exploratory post−hoc subgroup analysis of nine participants who voluntarily received KXA (self−controlled) showed significant reductions in LLS and AMS incidence, consistent with the trends observed in the main trial. This study demonstrates that KXA aerosol exhibits potential for preventing AMS during simulated high-altitude exposure. Future large-scale randomized controlled trials in authentic high-altitude environments are needed to validate its efficacy and elucidate its specific mechanisms of action.

## Data Availability

The raw data supporting the conclusions of this article will be made available by the authors, without undue reservation.

## Supplementary material

Supplementary Figure 1 Blood pressure trends in the KXA and placebo groups over time. **(a)**. Systolic Blood Pressure. **(b)**. Diastolic Blood Pressure. The blue line indicates hypobaric exposure with placebo, and the green line indicates hypobaric exposure with KXA.

Supplementary Figure 2 Temporal trends in physiological and cognitive outcomes during the two 4000m exposures (self-controlled). **(a)**. Peripheral oxygen saturation (SpO_2_). **(b)**. heart rate (HR), and **(c)**. headache severity (VAS) assessed hourly. Picture Memory Test (PMT, **(d)**) and Symbol-Digit Modalities Test **(e)** scores at baseline, 1h, and 6h. The blue line indicates the first time entering the chamber (Placebo), and the green line indicates second time entering the chamber (KXA).

Supplementary Figure 3 Comparison of global and tissue-specific cerebral blood flow (CBF) across experimental conditions (self-controlled). Bar graphs displaying the mean CBF values for **(a)** Whole Brain, **(b)** Gray Matter, and **(c)** White Matter. Measurements were taken at three time points: Baseline (BL), Post-Control Exposure (T1), and Post-KXA Intervention Exposure (T2). Error bars represent standard deviations. ∗ indicates a significant difference (*p* < 0.05).

Supplementary Figure 4 Regional cerebral blood flow (CBF) alterations and their distribution across functional networks (self-controlled). **(a)** Regions with CBF changes between entering the chambers. **(b)** Proportion of Significant ROIs per Network (%) calculated for the regions exhibiting CBF changes between entering the chambers. **(c)** Hypobaric hypoxic exposure with placebo vs. Baseline. **(d)** Proportion of Significant ROIs per Network (%) calculated for the comparison between hypobaric hypoxic exposure with placebo and baseline. **(e)** Hypobaric hypoxic exposure with KXA vs. Baseline. **(f)** Proportion of Significant ROIs per Network (%) calculated for the comparison between hypobaric hypoxic exposure with KXA and baseline. Cool colors represent decreased CBF, and warm colors represent increased CBF. The significance threshold was set at *p* < 0.05 (uncorrected).

Supplementary Table 1 Regional cerebral blood flow (CBF) differences between the KXA and placebo groups after hypobaric hypoxic exposure (regions with uncorrected p < 0.05).

Supplementary Table 2 Full list of regional cerebral blood flow (CBF) differences between the KXA and placebo groups after hypobaric hypoxic exposure (all ROIs, uncorrected).

Supplementary Table 3 ROI-level repeated-measures ANOVA results of CBF for the exploratory voluntary additional session (self-controlled, pre-placebo vs post-KXA).

Supplementary Table 4 *Post hoc* ROI-level comparisons of CBF between sessions in the exploratory voluntary additional session (self-controlled).
