# Supplementary material for: Effects of Kuanxiong aerosol for preventing acute mountain sickness: an exploratory small-sample randomized, placebo-controlled trial
Source: Front Physiol. 2026 Jul 21;17:1834301. doi: 10.3389/fphys.2026.1834301 (PMC13433183; doi:10.3389/fphys.2026.1834301)
Supplement: Supplementary file 1 [file Table1.docx]

**Supplementary Table 1. Regional cerebral blood flow (CBF) differences between the KXA and placebo groups after hypobaric hypoxic exposure (regions with uncorrected p < 0.05)**

| **ROI name** | **Coordinate** | | | ***T* value** | ***P* value** |
| --- | --- | --- | --- | --- | --- |
|  | x | y | z |  |  |
| **LH** |  |  |  |  |  |
| **Limbic** |  |  |  |  |  |
| OFC_1 | -12 | 25 | -21 | -3.5386 | 0.0030* |
| OFC_3 | -10 | 47 | -21 | -2.4106 | 0.0292* |
| **RH** |  |  |  |  |  |
| **Cont** |  |  |  |  |  |
| PFCmp_1 | -4 | 28 | 47 | -2.1971 | 0.0441* |

Note: * indicates p < 0.05 (uncorrected for multiple comparisons). Limbic, Limbic network; Cont, Frontoparietal Control network. LH and RH indicate the left and right hemispheres, respectively.
