# Supplementary material for: Effects of Kuanxiong aerosol for preventing acute mountain sickness: an exploratory small-sample randomized, placebo-controlled trial
Source: Front Physiol. 2026 Jul 21;17:1834301. doi: 10.3389/fphys.2026.1834301 (PMC13433183; doi:10.3389/fphys.2026.1834301)
Supplement: Supplementary file 2 [file Table2.docx]

**Supplementary Table 2. Full list of regional cerebral blood flow (CBF) differences between the KXA and placebo groups after hypobaric hypoxic exposure (all ROIs, uncorrected)**

| **ROI name** | **Coordinate** | | | ***F* value** | ***P* value** | **ROI name** | **Coordinate** | | | ***F* value** | ***p* value** |
| --- | --- | --- | --- | --- | --- | --- | --- | --- | --- | --- | --- |
|  | x | y | z |  |  |  | x | y | z |  |  |
| **LH** |  |  |  |  |  | **RH** |  |  |  |  |  |
| **Vis** |  |  |  |  |  | **Vis** |  |  |  |  |  |
| 1 | -33 | -42 | -21 | -0.8750 | 0.3954 | 1 | 34 | -37 | -23 | -0.8850 | 0.3901 |
| 2 | -30 | -33 | -18 | -1.9787 | 0.0665 | 2 | 31 | -31 | -18 | -1.3284 | 0.2039 |
| 3 | -36 | -62 | -17 | -0.8365 | 0.4160 | 3 | 36 | -53 | -17 | -1.0144 | 0.3265 |
| 4 | -24 | -55 | -8 | -1.2104 | 0.2449 | 4 | 37 | -73 | -16 | -0.8492 | 0.4091 |
| 5 | -23 | -73 | -10 | -0.5870 | 0.5659 | 5 | 26 | -52 | -9 | -1.0144 | 0.3265 |
| 6 | -36 | -81 | -16 | -0.5609 | 0.5832 | 6 | 23 | -74 | -11 | -1.2107 | 0.2447 |
| 7 | -18 | -37 | -12 | -1.3962 | 0.1830 | 7 | 18 | -36 | -12 | -1.0672 | 0.3028 |
| 8 | -45 | -70 | -8 | -0.7630 | 0.4573 | 8 | 20 | -88 | -13 | -0.6657 | 0.5157 |
| 9 | -17 | -86 | -15 | -0.6724 | 0.5115 | 9 | 50 | -64 | -9 | -0.4139 | 0.6848 |
| 10 | -12 | -62 | -5 | -1.5862 | 0.1336 | 10 | 42 | -84 | -12 | -0.5519 | 0.5891 |
| 11 | -7 | -76 | -6 | -1.4971 | 0.1551 | 11 | 9 | -72 | -5 | -1.1680 | 0.2610 |
| 12 | -24 | -97 | -12 | -0.0539 | 0.9577 | 12 | 13 | -58 | -3 | -1.1642 | 0.2625 |
| 13 | -13 | -43 | -5 | -0.2890 | 0.7765 | 13 | 25 | -97 | -10 | -0.7334 | 0.4746 |
| 14 | -41 | -87 | -3 | -0.5273 | 0.6057 | 14 | 18 | -45 | -3 | -1.3751 | 0.1893 |
| 15 | -7 | -98 | -7 | 0.2851 | 0.7795 | 15 | 8 | -92 | -2 | -0.5517 | 0.5893 |
| 16 | -14 | -57 | 1 | -1.7033 | 0.1091 | 16 | 48 | -66 | 4 | -0.5642 | 0.5810 |
| 17 | -46 | -73 | 6 | -0.4706 | 0.6447 | 17 | 14 | -46 | 4 | -1.6257 | 0.1248 |
| 18 | -5 | -88 | 2 | -0.1484 | 0.8840 | 18 | 35 | -89 | 2 | -0.3242 | 0.7503 |
| 19 | -24 | -96 | 6 | -0.4701 | 0.6451 | 19 | 9 | -74 | 9 | -0.9900 | 0.3379 |
| 20 | -13 | -49 | 4 | -1.4207 | 0.1758 | 20 | 22 | -59 | 6 | -1.3638 | 0.1927 |
| 21 | -7 | -74 | 9 | -0.3847 | 0.7059 | 21 | 24 | -99 | 7 | -0.1471 | 0.8850 |
| 22 | -19 | -65 | 7 | -1.2949 | 0.2149 | 22 | 43 | -79 | 10 | -0.7020 | 0.4934 |
| 23 | -39 | -84 | 14 | -0.3235 | 0.7508 | 23 | 13 | -94 | 19 | -0.8603 | 0.4032 |
| 24 | -11 | -97 | 17 | -0.0936 | 0.9266 | 24 | 16 | -66 | 19 | -1.5354 | 0.1455 |
| 25 | -3 | -84 | 24 | -0.6845 | 0.5041 | 25 | 5 | -80 | 24 | -0.9480 | 0.3581 |
| 26 | -12 | -71 | 20 | -0.1464 | 0.8856 | 26 | 27 | -87 | 21 | -0.5099 | 0.6176 |
| 27 | -25 | -85 | 21 | -0.6400 | 0.5318 | 27 | 36 | -79 | 24 | -0.3374 | 0.7405 |
| 28 | -32 | -84 | 27 | -0.4988 | 0.6251 | 28 | 14 | -78 | 34 | -0.6583 | 0.5203 |
| 29 | -16 | -89 | 33 | -0.6035 | 0.5552 | 29 | 16 | -87 | 36 | -0.9451 | 0.3596 |
| 30 | -12 | -81 | 36 | -0.6800 | 0.5069 | 30 | 29 | -78 | 37 | -0.0933 | 0.9269 |
| 31 | -21 | -79 | 45 | 0.3537 | 0.7285 | **SomMot** |  |  |  |  |  |
| **SomMot** |  |  |  |  |  | 1 | 53 | 3 | -6 | -0.3659 | 0.7196 |
| 1 | -50 | -9 | 0 | -0.7572 | 0.4607 | 2 | 62 | -19 | 0 | -0.8619 | 0.4023 |
| 2 | -56 | -22 | 8 | -1.2456 | 0.2320 | 3 | 53 | -14 | 6 | -0.4239 | 0.6777 |
| 3 | -36 | -24 | 10 | -0.7121 | 0.4873 | 4 | 39 | -19 | 5 | 0.2252 | 0.8249 |
| 4 | -38 | -9 | 13 | -0.4023 | 0.6932 | 5 | 37 | -8 | 14 | 0.4585 | 0.6532 |
| 5 | -36 | -26 | 19 | -0.8273 | 0.4211 | 6 | 35 | -21 | 14 | -0.3621 | 0.7223 |
| 6 | -59 | -37 | 16 | -0.2726 | 0.7889 | 7 | 60 | -24 | 11 | -0.0012 | 0.9990 |
| 7 | -41 | -35 | 14 | 0.2968 | 0.7707 | 8 | 65 | -34 | 11 | -0.5021 | 0.6229 |
| 8 | -49 | -13 | 14 | -0.0707 | 0.9446 | 9 | 41 | -13 | 18 | 0.8406 | 0.4138 |
| 9 | -60 | -2 | 11 | 0.3862 | 0.7048 | 10 | 41 | -29 | 18 | 0.5348 | 0.6006 |
| 10 | -48 | -24 | 18 | 0.3593 | 0.7244 | 11 | 50 | -10 | 13 | 0.0687 | 0.9461 |
| 11 | -61 | -18 | 19 | -1.4444 | 0.1692 | 12 | 59 | 1 | 10 | 0.2665 | 0.7935 |
| 12 | -59 | -2 | 23 | 0.4173 | 0.6824 | 13 | 49 | -21 | 19 | 0.0106 | 0.9917 |
| 13 | -63 | -10 | 31 | -0.4127 | 0.6857 | 14 | 62 | -12 | 15 | 0.0271 | 0.9787 |
| 14 | -53 | -9 | 30 | 0.3964 | 0.6974 | 15 | 61 | 6 | 30 | 0.7987 | 0.4369 |
| 15 | -55 | -20 | 41 | -1.0243 | 0.3219 | 16 | 60 | -5 | 27 | 0.4425 | 0.6644 |
| 16 | -51 | -7 | 43 | 0.1891 | 0.8526 | 17 | 61 | -14 | 30 | -0.2562 | 0.8013 |
| 17 | -48 | -16 | 40 | -0.1410 | 0.8897 | 18 | 52 | -6 | 37 | 0.3999 | 0.6948 |
| 18 | -8 | -15 | 47 | -0.0485 | 0.9619 | 19 | 54 | -17 | 40 | -0.7258 | 0.4791 |
| 19 | -41 | -14 | 47 | -0.7762 | 0.4497 | 20 | 11 | -17 | 41 | 0.5570 | 0.5857 |
| 20 | -49 | -17 | 54 | -1.6875 | 0.1122 | 21 | 52 | -13 | 49 | -0.5114 | 0.6165 |
| 21 | -48 | -29 | 58 | -1.1453 | 0.2700 | 22 | 44 | -10 | 49 | 0.8347 | 0.4170 |
| 22 | -39 | -25 | 53 | -0.6994 | 0.4950 | 23 | 49 | -26 | 56 | -0.7144 | 0.4860 |
| 23 | -9 | -38 | 54 | -0.8283 | 0.4205 | 24 | 7 | -10 | 51 | -0.1742 | 0.8640 |
| 24 | -4 | -25 | 56 | -0.9895 | 0.3381 | 25 | 43 | -21 | 54 | -0.0450 | 0.9647 |
| 25 | -4 | -9 | 59 | 0.1769 | 0.8619 | 26 | 37 | -20 | 64 | 0.1134 | 0.9112 |
| 26 | -36 | -19 | 65 | -0.9789 | 0.3431 | 27 | 32 | -34 | 63 | 0.0308 | 0.9759 |
| 27 | -30 | -46 | 63 | -0.2790 | 0.7840 | 28 | 31 | -41 | 64 | 0.0267 | 0.9790 |
| 28 | -32 | -29 | 63 | -1.1924 | 0.2516 | 29 | 34 | -27 | 61 | 0.3706 | 0.7161 |
| 29 | -30 | -38 | 65 | -0.3806 | 0.7089 | 30 | 4 | -25 | 58 | -0.8738 | 0.3960 |
| 30 | -23 | -11 | 65 | -0.6056 | 0.5538 | 31 | 29 | -11 | 65 | -0.9831 | 0.3411 |
| 31 | -19 | -24 | 67 | -0.2438 | 0.8107 | 32 | 9 | -40 | 68 | -1.3816 | 0.1873 |
| 32 | -9 | -42 | 70 | -0.4495 | 0.6595 | 33 | 21 | -24 | 67 | 0.2631 | 0.7960 |
| 33 | -4 | -25 | 69 | -1.7319 | 0.1038 | 34 | 22 | -29 | 68 | -0.7754 | 0.4502 |
| 34 | -14 | -11 | 73 | -1.4872 | 0.1577 | 35 | 22 | -35 | 71 | -0.6740 | 0.5105 |
| 35 | -19 | -31 | 69 | -1.3983 | 0.1824 | 36 | 17 | -6 | 69 | -0.8015 | 0.4354 |
| 36 | -19 | -40 | 72 | -1.0192 | 0.3242 | 37 | 16 | -47 | 74 | -0.0263 | 0.9794 |
| 37 | -12 | -27 | 73 | -1.1623 | 0.2633 | 38 | 5 | -22 | 72 | -1.5011 | 0.1541 |
| **DorsAttn** |  |  |  |  |  | 39 | 17 | -18 | 73 | -1.8563 | 0.0832 |
| Post_1 | -45 | -42 | -21 | -0.7786 | 0.4483 | 40 | 13 | -33 | 76 | -1.3243 | 0.2052 |
| Post_2 | -49 | -56 | -15 | -0.5847 | 0.5674 | **DorsAttn** |  |  |  |  |  |
| Post_3 | -55 | -62 | -1 | -0.4863 | 0.6338 | Post_1 | 50 | -49 | -18 | -0.4758 | 0.6411 |
| Post_4 | -48 | -65 | 15 | -0.5555 | 0.5867 | Post_2 | 59 | -55 | -2 | -0.2263 | 0.8240 |
| Post_5 | -26 | -70 | 31 | -0.3434 | 0.7361 | Post_3 | 54 | -56 | 12 | 0.0105 | 0.9918 |
| Post_6 | -55 | -32 | 45 | -0.7841 | 0.4452 | Post_4 | 45 | -75 | 31 | 0.3485 | 0.7323 |
| Post_7 | -23 | -65 | 46 | 0.0763 | 0.9402 | Post_5 | 32 | -66 | 35 | -0.0206 | 0.9838 |
| Post_8 | -46 | -29 | 44 | -0.5510 | 0.5898 | Post_6 | 57 | -23 | 44 | -0.7844 | 0.4450 |
| Post_9 | -33 | -46 | 41 | -0.7293 | 0.4770 | Post_7 | 19 | -79 | 50 | -0.3140 | 0.7579 |
| Post_10 | -29 | -58 | 50 | 0.2506 | 0.8055 | Post_8 | 44 | -37 | 50 | -0.5201 | 0.6106 |
| Post_11 | -39 | -37 | 49 | -0.6368 | 0.5339 | Post_9 | 45 | -28 | 42 | -0.2124 | 0.8347 |
| Post_12 | -36 | -52 | 56 | -0.7253 | 0.4794 | Post_10 | 31 | -64 | 53 | 0.3240 | 0.7504 |
| Post_13 | -15 | -71 | 57 | 0.0702 | 0.9450 | Post_11 | 36 | -44 | 45 | -0.8132 | 0.4289 |
| Post_14 | -29 | -61 | 62 | -0.1721 | 0.8657 | Post_12 | 21 | -69 | 53 | 0.2033 | 0.8417 |
| Post_15 | -7 | -59 | 63 | 0.5203 | 0.6104 | Post_13 | 35 | -36 | 51 | -0.2650 | 0.7946 |
| Post_16 | -20 | -57 | 66 | -0.0071 | 0.9944 | Post_14 | 34 | -50 | 54 | -0.4827 | 0.6363 |
| Post_17 | -13 | -50 | 72 | 0.6027 | 0.5557 | Post_15 | 8 | -71 | 53 | -0.8321 | 0.4184 |
| FEF_1 | -40 | -3 | 51 | 0.1375 | 0.8925 | Post_16 | 27 | -58 | 61 | 0.2430 | 0.8113 |
| FEF_2 | -25 | -1 | 55 | -0.1652 | 0.8710 | Post_17 | 7 | -54 | 59 | -0.5039 | 0.6217 |
| FEF_3 | -30 | -8 | 52 | -0.1816 | 0.8583 | Post_18 | 14 | -64 | 65 | 0.2488 | 0.8069 |
| FEF_4 | -21 | 5 | 65 | -0.3808 | 0.7087 | Post_19 | 24 | -50 | 68 | -0.3781 | 0.7106 |
| PrCv_1 | -49 | 6 | 26 | 0.8486 | 0.4095 | FEF_1 | 39 | -3 | 53 | 0.0632 | 0.9504 |
| PrCv_2 | -50 | 3 | 38 | 0.8782 | 0.3937 | FEF_2 | 27 | -3 | 52 | 0.3685 | 0.7177 |
| **SalVentAttn** |  |  |  |  |  | FEF_3 | 25 | -3 | 64 | -0.6868 | 0.5027 |
| ParOper_1 | -55 | -32 | 22 | -0.3648 | 0.7204 | PrCv_1 | 49 | 8 | 25 | 0.6122 | 0.5496 |
| ParOper_2 | -58 | -44 | 27 | -0.1800 | 0.8596 | **SalVentAttn** |  |  |  |  |  |
| ParOper_3 | -61 | -23 | 33 | -0.7635 | 0.4570 | TempOccPar_1 | 59 | -46 | 7 | -0.2242 | 0.8256 |
| ParOper_4 | -61 | -36 | 33 | -0.1815 | 0.8584 | TempOccPar_2 | 51 | -41 | 13 | 0.1033 | 0.9191 |
| TempOcc_1 | -57 | -54 | 10 | -0.7353 | 0.4735 | TempOccPar_3 | 62 | -40 | 22 | -0.1495 | 0.8831 |
| FrOperIns_1 | -44 | 5 | -17 | -1.0223 | 0.3228 | TempOccPar_4 | 58 | -31 | 24 | 0.3942 | 0.6990 |
| FrOperIns_2 | -39 | 2 | -4 | -0.1954 | 0.8477 | TempOccPar_5 | 60 | -22 | 22 | -0.2684 | 0.7920 |
| FrOperIns_3 | -33 | 25 | -1 | 1.1187 | 0.2809 | TempOccPar_6 | 63 | -26 | 38 | 0.1281 | 0.8997 |
| FrOperIns_4 | -40 | -15 | -2 | -0.2729 | 0.7887 | TempOccPar_7 | 62 | -37 | 37 | -0.4192 | 0.6810 |
| FrOperIns_5 | -33 | 19 | 8 | 1.1572 | 0.2653 | PrC_1 | 51 | 3 | 41 | 1.3372 | 0.2011 |
| FrOperIns_6 | -36 | 4 | 11 | 0.6328 | 0.5364 | FrOperIns_1 | 40 | 5 | -15 | -0.1948 | 0.8482 |
| FrOperIns_7 | -43 | 12 | 2 | 0.6515 | 0.5246 | FrOperIns_2 | 41 | 8 | -3 | 0.2781 | 0.7847 |
| FrOperIns_8 | -50 | 1 | 5 | 1.2485 | 0.2310 | FrOperIns_3 | 40 | -10 | -4 | 0.0826 | 0.9353 |
| FrOperIns_9 | -52 | 9 | 13 | 0.7944 | 0.4393 | FrOperIns_4 | 39 | -2 | 6 | -0.1856 | 0.8553 |
| PFCl_1 | -29 | 43 | 30 | 0.3254 | 0.7494 | FrOperIns_5 | 37 | 23 | 5 | 1.5462 | 0.1429 |
| Med_1 | -6 | 22 | 31 | 0.1888 | 0.8527 | FrOperIns_6 | 38 | 7 | 11 | 0.6015 | 0.5565 |
| Med_2 | -7 | 0 | 41 | -0.0325 | 0.9745 | FrOperIns_7 | 49 | 5 | 3 | 0.4557 | 0.6551 |
| Med_3 | -11 | -27 | 41 | -0.5726 | 0.5754 | FrOperIns_8 | 54 | 12 | 12 | 0.8987 | 0.3830 |
| Med_4 | -5 | 9 | 48 | -0.3776 | 0.7110 | PFCl_1 | 33 | 45 | 28 | 0.6189 | 0.5453 |
| Med_5 | -13 | -41 | 47 | -1.1392 | 0.2725 | Med_1 | 7 | 19 | 35 | 0.6421 | 0.5305 |
| Med_6 | -6 | -49 | 57 | -0.1033 | 0.9191 | Med_2 | 7 | 2 | 43 | 0.8371 | 0.4156 |
| Med_7 | -8 | -3 | 71 | -0.6282 | 0.5393 | Med_3 | 12 | -34 | 43 | -0.6813 | 0.5061 |
| **Limbic** |  |  |  |  |  | Med_4 | 6 | 11 | 58 | 0.1736 | 0.8645 |
| OFC_1 | -12 | 25 | -21 | 3.5386 | 0.0030* | Med_5 | 10 | -43 | 53 | -0.6225 | 0.5429 |
| OFC_2 | -24 | 23 | -20 | 0.3699 | 0.7166 | Med_6 | 11 | -32 | 50 | -0.6621 | 0.5180 |
| OFC_3 | -10 | 47 | -21 | 2.4106 | 0.0292* | Med_7 | 7 | -2 | 67 | -0.1904 | 0.8515 |
| OFC_4 | -4 | 23 | -19 | 1.2945 | 0.2151 | Med_8 | 16 | 7 | 69 | 0.4993 | 0.6248 |
| OFC_5 | -15 | 65 | -8 | 0.5805 | 0.5702 | **Limbic** |  |  |  |  |  |
| TempPole_1 | -37 | -5 | -42 | -0.4498 | 0.6593 | OFC_1 | 13 | 24 | -21 | 0.3164 | 0.7561 |
| TempPole_2 | -25 | 6 | -39 | 0.0598 | 0.9531 | OFC_2 | 23 | 22 | -21 | -1.1276 | 0.2772 |
| TempPole_3 | -26 | -9 | -33 | -0.5643 | 0.5809 | OFC_3 | 8 | 47 | -23 | 1.1123 | 0.2835 |
| TempPole_4 | -54 | -21 | -31 | 0.4073 | 0.6895 | OFC_4 | 20 | 43 | -18 | 0.5622 | 0.5823 |
| TempPole_5 | -40 | -21 | -27 | -0.2334 | 0.8186 | OFC_5 | 5 | 22 | -21 | 0.7083 | 0.4896 |
| TempPole_6 | -44 | 13 | -34 | -0.7779 | 0.4487 | OFC_6 | 9 | 63 | -14 | 0.7110 | 0.4880 |
| TempPole_7 | -32 | 12 | -29 | -0.1175 | 0.9081 | TempPole_1 | 28 | -1 | -40 | -0.4145 | 0.6843 |
| TempPole_8 | -21 | -21 | -26 | -0.8522 | 0.4075 | TempPole_2 | 49 | -7 | -39 | -0.7781 | 0.4486 |
| **Cont** |  |  |  |  |  | TempPole_3 | 37 | 17 | -38 | 1.1384 | 0.2728 |
| Par_1 | -29 | -74 | 42 | -0.1916 | 0.8506 | TempPole_4 | 39 | -15 | -31 | -0.4405 | 0.6659 |
| Par_2 | -58 | -42 | 45 | -0.0103 | 0.9919 | TempPole_5 | 29 | 12 | -30 | 0.3034 | 0.7657 |
| Par_3 | -53 | -50 | 45 | 0.5342 | 0.6011 | TempPole_6 | 50 | -28 | -26 | -0.5982 | 0.5586 |
| Par_4 | -35 | -62 | 48 | 0.0280 | 0.9780 | TempPole_7 | 23 | -18 | -27 | -0.8887 | 0.3882 |
| Par_5 | -42 | -52 | 49 | -0.5164 | 0.6131 | **Cont** |  |  |  |  |  |
| Par_6 | -45 | -41 | 47 | -0.7900 | 0.4418 | Par_1 | 54 | -53 | 44 | 0.1333 | 0.8957 |
| Temp_1 | -60 | -49 | -10 | -0.3744 | 0.7134 | Par_2 | 56 | -41 | 48 | 0.4225 | 0.6787 |
| OFC_1 | -27 | 49 | -14 | 1.4639 | 0.1639 | Par_3 | 35 | -71 | 47 | 0.2709 | 0.7902 |
| PFCl_1 | -42 | 49 | -6 | 1.3136 | 0.2087 | Par_4 | 54 | -33 | 51 | -0.1167 | 0.9087 |
| PFCl_2 | -48 | 35 | 10 | 0.2470 | 0.8083 | Par_5 | 47 | -44 | 46 | 0.1435 | 0.8878 |
| PFCl_3 | -38 | 49 | 11 | 0.4002 | 0.6947 | Par_6 | 41 | -55 | 48 | 0.9451 | 0.3596 |
| PFCl_4 | -28 | 57 | 13 | 0.2216 | 0.8276 | Temp_1 | 62 | -28 | -20 | 1.3456 | 0.1984 |
| PFCl_5 | -42 | 38 | 22 | -0.2060 | 0.8395 | Temp_2 | 63 | -42 | -11 | 0.2438 | 0.8107 |
| PFCl_6 | -45 | 20 | 27 | 0.4215 | 0.6794 | PFCv_1 | 34 | 21 | -8 | -0.1736 | 0.8645 |
| PFCl_7 | -39 | 7 | 34 | 0.1310 | 0.8975 | PFCl_1 | 28 | 55 | -14 | 2.0697 | 0.0562 |
| PFCl_8 | -36 | 32 | 38 | -0.3489 | 0.7320 | PFCl_2 | 42 | 51 | -6 | 1.8322 | 0.0869 |
| PFCv_1 | -34 | 16 | -8 | 0.6718 | 0.5119 | PFCl_3 | 27 | 59 | 3 | 1.1188 | 0.2808 |
| pCun_1 | -9 | -77 | 45 | -0.7913 | 0.4411 | PFCl_4 | 49 | 40 | 5 | -0.0513 | 0.9598 |
| pCun_2 | -5 | -64 | 52 | -0.8196 | 0.4253 | PFCl_5 | 42 | 46 | 14 | 0.5123 | 0.6159 |
| Cing_1 | -3 | 5 | 29 | -0.4889 | 0.6320 | PFCl_6 | 50 | 30 | 18 | 0.8380 | 0.4152 |
| Cing_2 | -4 | -22 | 29 | -0.6533 | 0.5234 | PFCl_7 | 48 | 18 | 23 | 1.1128 | 0.2833 |
| PFCmp_1 | -4 | 28 | 47 | -0.3471 | 0.7333 | PFCl_8 | 25 | 54 | 25 | 0.6948 | 0.4978 |
| **Default** |  |  |  |  |  | PFCl_9 | 47 | 29 | 28 | 0.7974 | 0.4377 |
| Temp_1 | -54 | -2 | -30 | -1.5652 | 0.1384 | PFCl_10 | 39 | 11 | 34 | 1.3513 | 0.1966 |
| Temp_2 | -62 | -18 | -21 | 0.3592 | 0.7245 | PFCl_11 | 39 | 33 | 38 | 0.8102 | 0.4305 |
| Temp_3 | -60 | -36 | -18 | 0.9351 | 0.3646 | PFCl_12 | 45 | 19 | 44 | 1.2207 | 0.2410 |
| Temp_4 | -57 | -9 | -14 | -1.8877 | 0.0786 | PFCl_13 | 43 | 7 | 51 | 1.4427 | 0.1697 |
| Temp_5 | -53 | 6 | -11 | -0.1767 | 0.8621 | PFCl_14 | 34 | 15 | 56 | 0.2118 | 0.8351 |
| Temp_6 | -61 | -35 | -3 | -0.5649 | 0.5805 | PFCl_15 | 24 | 10 | 58 | 0.6793 | 0.5073 |
| Temp_7 | -52 | -22 | -6 | -0.5843 | 0.5677 | pCun_1 | 13 | -71 | 39 | -0.8016 | 0.4353 |
| Temp_8 | -61 | -13 | -3 | -1.5067 | 0.1527 | pCun_2 | 5 | -64 | 44 | -0.6351 | 0.5349 |
| Temp_9 | -62 | -32 | 5 | -0.5520 | 0.5891 | Cing_1 | 6 | -26 | 28 | 0.3485 | 0.7323 |
| Temp_10 | -52 | -43 | 5 | -0.8284 | 0.4205 | Cing_2 | 5 | 1 | 30 | 0.9161 | 0.3741 |
| Par_1 | -59 | -49 | 16 | -0.7289 | 0.4773 | PFCmp_1 | 8 | 35 | 25 | 2.1971 | 0.0441* |
| Par_2 | -45 | -58 | 21 | -0.2926 | 0.7738 | PFCmp_2 | 5 | 28 | 48 | 0.3252 | 0.7495 |
| Par_3 | -40 | -79 | 30 | -0.0804 | 0.9369 | **Default** |  |  |  |  |  |
| Par_4 | -47 | -64 | 31 | 0.0930 | 0.9271 | Par_1 | 55 | -46 | 19 | -0.2485 | 0.8071 |
| Par_5 | -57 | -55 | 30 | -0.5763 | 0.5730 | Par_2 | 48 | -64 | 22 | 0.3153 | 0.7569 |
| Par_6 | -41 | -72 | 43 | 0.0499 | 0.9608 | Par_3 | 53 | -53 | 26 | -0.0472 | 0.9630 |
| Par_7 | -49 | -60 | 47 | 0.1506 | 0.8823 | Par_4 | 55 | -45 | 33 | 0.2710 | 0.7901 |
| PFC_1 | -36 | 22 | -16 | -0.8963 | 0.3843 | Par_5 | 47 | -64 | 42 | 1.3625 | 0.1931 |
| PFC_2 | -36 | 37 | -13 | 0.6484 | 0.5265 | Temp_1 | 49 | 9 | -33 | -0.5802 | 0.5704 |
| PFC_3 | -5 | 55 | -10 | 0.3353 | 0.7420 | Temp_2 | 61 | -8 | -23 | -0.8046 | 0.4336 |
| PFC_4 | -6 | 35 | -9 | 1.2387 | 0.2345 | Temp_3 | 47 | 16 | -20 | 0.3458 | 0.7343 |
| PFC_5 | -46 | 32 | -10 | 0.1400 | 0.8905 | Temp_4 | 55 | -4 | -14 | -0.8830 | 0.3912 |
| PFC_6 | -28 | 58 | -1 | 1.1520 | 0.2674 | Temp_5 | 63 | -23 | -7 | 0.0501 | 0.9607 |
| PFC_7 | -48 | 28 | 0 | 0.7503 | 0.4647 | Temp_6 | 49 | -20 | -8 | -0.0218 | 0.9829 |
| PFC_8 | -6 | 59 | 7 | 0.6360 | 0.5344 | Temp_7 | 63 | -38 | 0 | 0.2203 | 0.8286 |
| PFC_9 | -6 | 45 | 6 | 0.8165 | 0.4270 | Temp_8 | 50 | -33 | 2 | 0.2397 | 0.8138 |
| PFC_10 | -53 | 19 | 11 | 0.6100 | 0.5510 | PFCv_1 | 35 | 23 | -18 | -0.1247 | 0.9024 |
| PFC_11 | -16 | 67 | 8 | 0.0712 | 0.9442 | PFCv_2 | 35 | 38 | -13 | -0.3839 | 0.7065 |
| PFC_12 | -5 | 34 | 21 | 0.3261 | 0.7488 | PFCv_3 | 48 | 32 | -8 | 0.4743 | 0.6421 |
| PFC_13 | -4 | 51 | 28 | -0.4696 | 0.6454 | PFCv_4 | 54 | 24 | 6 | 0.7259 | 0.4791 |
| PFC_14 | -14 | 58 | 31 | 0.1809 | 0.8589 | PFCdPFCm_1 | 5 | 41 | -11 | 0.9987 | 0.3338 |
| PFC_15 | -22 | 51 | 31 | -0.1618 | 0.8736 | PFCdPFCm_2 | 9 | 67 | 1 | 0.2304 | 0.8209 |
| PFC_16 | -25 | 28 | 43 | 1.3825 | 0.1871 | PFCdPFCm_3 | 7 | 42 | 4 | 1.0909 | 0.2925 |
| PFC_17 | -41 | 19 | 48 | 0.1896 | 0.8521 | PFCdPFCm_4 | 7 | 54 | 13 | 1.1430 | 0.2710 |
| PFC_18 | -8 | 43 | 51 | -0.8370 | 0.4158 | PFCdPFCm_5 | 17 | 65 | 16 | 0.1530 | 0.8804 |
| PFC_19 | -18 | 36 | 48 | 0.2449 | 0.8099 | PFCdPFCm_6 | 6 | 25 | 18 | 0.7063 | 0.4908 |
| PFC_20 | -42 | 7 | 48 | -0.1700 | 0.8673 | PFCdPFCm_7 | 6 | 58 | 29 | -0.1020 | 0.9201 |
| PFC_21 | -30 | 14 | 57 | -0.1332 | 0.8958 | PFCdPFCm_8 | 16 | 52 | 36 | 0.7265 | 0.4787 |
| PFC_22 | -22 | 20 | 52 | 0.6127 | 0.5492 | PFCdPFCm_9 | 5 | 44 | 40 | -0.1804 | 0.8593 |
| PFC_23 | -13 | 24 | 61 | -0.3097 | 0.7611 | PFCdPFCm_10 | 26 | 34 | 39 | 1.2404 | 0.2339 |
| PFC_24 | -6 | 10 | 65 | -0.6159 | 0.5472 | PFCdPFCm_11 | 14 | 39 | 52 | -0.0238 | 0.9813 |
| pCunPCC_1 | -8 | -52 | 9 | -1.5162 | 0.1503 | PFCdPFCm_12 | 24 | 26 | 51 | 0.8723 | 0.3968 |
| pCunPCC_2 | -13 | -61 | 19 | -1.2878 | 0.2173 | PFCdPFCm_13 | 12 | 20 | 63 | -0.0463 | 0.9637 |
| pCunPCC_3 | -4 | -53 | 20 | -1.0230 | 0.3225 | pCunPCC_1 | 12 | -55 | 15 | -1.2347 | 0.2359 |
| pCunPCC_4 | -6 | -41 | 24 | -0.5929 | 0.5621 | pCunPCC_2 | 7 | -44 | 20 | -0.5453 | 0.5935 |
| pCunPCC_5 | -10 | -70 | 32 | -0.7404 | 0.4705 | pCunPCC_3 | 17 | -63 | 28 | -1.0701 | 0.3015 |
| pCunPCC_6 | -5 | -60 | 30 | -0.9472 | 0.3586 | pCunPCC_4 | 6 | -52 | 23 | -0.6949 | 0.4977 |
| pCunPCC_7 | -7 | -44 | 32 | -1.1476 | 0.2691 | pCunPCC_5 | 5 | -63 | 31 | -0.7706 | 0.4529 |
| pCunPCC_8 | -4 | -34 | 38 | -0.5006 | 0.6239 | pCunPCC_6 | 7 | -39 | 35 | -0.6080 | 0.5523 |
| pCunPCC_9 | -3 | -15 | 37 | -0.2238 | 0.8259 | pCunPCC_7 | 4 | -20 | 37 | 0.4675 | 0.6469 |
| pCunPCC_10 | -3 | -68 | 41 | -0.9073 | 0.3786 | pCunPCC_8 | 10 | -53 | 35 | -0.4079 | 0.6891 |

Note: * indicates p < 0.05 (uncorrected for multiple comparisons). Vis, Visual network; SomMot, Somatomotor network; DorsAttn, Dorsal Attention network; SalVentAttn, Salience/Ventral Attention network; Limbic, Limbic network; Cont, Frontoparietal Control network; Default, Default Mode network. LH and RH indicate the left and right hemispheres, respectively.
