# Supplementary material for: Effects of Kuanxiong aerosol for preventing acute mountain sickness: an exploratory small-sample randomized, placebo-controlled trial
Source: Front Physiol. 2026 Jul 21;17:1834301. doi: 10.3389/fphys.2026.1834301 (PMC13433183; doi:10.3389/fphys.2026.1834301)
Supplement: Supplementary file 3 [file Table3.docx]

**Supplementary Table 3. ROI‑level repeated‑measures ANOVA results of CBF for the exploratory voluntary additional session (self‑controlled, pre‑placebo vs post‑KXA)**

| **ROI name** | **Coordinate** | | | ***F* value** | ***P* value** | **ROI name** | **Coordinate** | | | ***F* value** | ***p* value** |
| --- | --- | --- | --- | --- | --- | --- | --- | --- | --- | --- | --- |
|  | x | y | z |  |  |  | x | y | z |  |  |
| **LH** |  |  |  |  |  | **RH** |  |  |  |  |  |
| **Vis** |  |  |  |  |  | **Vis** |  |  |  |  |  |
| 1 | -33 | -42 | -21 | 0.3284 | 0.7264 | 1 | 34 | -37 | -23 | 0.4491 | 0.6485 |
| 2 | -30 | -33 | -18 | 0.8367 | 0.4569 | 2 | 31 | -31 | -18 | 0.4796 | 0.6304 |
| 3 | -36 | -62 | -17 | 1.1935 | 0.3367 | 3 | 36 | -53 | -17 | 2.2275 | 0.1504 |
| 4 | -24 | -55 | -8 | 0.5353 | 0.5988 | 4 | 37 | -73 | -16 | 2.6519 | 0.1112 |
| 5 | -23 | -73 | -10 | 0.9183 | 0.4255 | 5 | 26 | -52 | -9 | 1.3268 | 0.3016 |
| 6 | -36 | -81 | -16 | 0.6143 | 0.5572 | 6 | 23 | -74 | -11 | 1.1871 | 0.3385 |
| 7 | -18 | -37 | -12 | 0.5932 | 0.5680 | 7 | 18 | -36 | -12 | 0.8503 | 0.4515 |
| 8 | -45 | -70 | -8 | 1.2466 | 0.3222 | 8 | 20 | -88 | -13 | 2.1031 | 0.1648 |
| 9 | -17 | -86 | -15 | 1.0715 | 0.3731 | 9 | 50 | -64 | -9 | 2.5684 | 0.1179 |
| 10 | -12 | -62 | -5 | 0.2313 | 0.7970 | 10 | 42 | -84 | -12 | 2.2942 | 0.1433 |
| 11 | -7 | -76 | -6 | 0.8414 | 0.4550 | 11 | 9 | -72 | -5 | 0.5364 | 0.5982 |
| 12 | -24 | -97 | -12 | 1.4193 | 0.2797 | 12 | 13 | -58 | -3 | 0.5891 | 0.5701 |
| 13 | -13 | -43 | -5 | 0.2779 | 0.7621 | 13 | 25 | -97 | -10 | 3.1157 | 0.0813 |
| 14 | -41 | -87 | -3 | 0.6275 | 0.5506 | 14 | 18 | -45 | -3 | 0.2940 | 0.7505 |
| 15 | -7 | -98 | -7 | 0.5621 | 0.5843 | 15 | 8 | -92 | -2 | 0.5035 | 0.6166 |
| 16 | -14 | -57 | 1 | 0.9828 | 0.4025 | 16 | 48 | -66 | 4 | 3.7561 | 0.0541 |
| 17 | -46 | -73 | 6 | 2.2351 | 0.1496 | 17 | 14 | -46 | 4 | 1.0338 | 0.3853 |
| 18 | -5 | -88 | 2 | 0.4407 | 0.6536 | 18 | 35 | -89 | 2 | 3.9532 | 0.0480* |
| 19 | -24 | -96 | 6 | 3.7193 | 0.0553 | 19 | 9 | -74 | 9 | 0.4646 | 0.6393 |
| 20 | -13 | -49 | 4 | 0.2684 | 0.7690 | 20 | 22 | -59 | 6 | 1.1173 | 0.3589 |
| 21 | -7 | -74 | 9 | 0.1984 | 0.8227 | 21 | 24 | -99 | 7 | 4.6923 | 0.0312* |
| 22 | -19 | -65 | 7 | 0.2355 | 0.7938 | 22 | 43 | -79 | 10 | 3.8878 | 0.0499* |
| 23 | -39 | -84 | 14 | 2.0028 | 0.1776 | 23 | 13 | -94 | 19 | 2.0969 | 0.1656 |
| 24 | -11 | -97 | 17 | 0.8855 | 0.4378 | 24 | 16 | -66 | 19 | 1.4577 | 0.2712 |
| 25 | -3 | -84 | 24 | 0.3417 | 0.7173 | 25 | 5 | -80 | 24 | 0.9497 | 0.4141 |
| 26 | -12 | -71 | 20 | 0.4745 | 0.6334 | 26 | 27 | -87 | 21 | 2.5749 | 0.1174 |
| 27 | -25 | -85 | 21 | 1.6828 | 0.2269 | 27 | 36 | -79 | 24 | 3.1624 | 0.0789 |
| 28 | -32 | -84 | 27 | 1.8728 | 0.1959 | 28 | 14 | -78 | 34 | 1.5368 | 0.2546 |
| 29 | -16 | -89 | 33 | 0.0366 | 0.9642 | 29 | 16 | -87 | 36 | 2.2573 | 0.1472 |
| 30 | -12 | -81 | 36 | 0.3196 | 0.7325 | 30 | 29 | -78 | 37 | 2.6843 | 0.1088 |
| 31 | -21 | -79 | 45 | 1.0696 | 0.3737 | **SomMot** |  |  |  |  |  |
| **SomMot** |  |  |  |  |  | 1 | 53 | 3 | -6 | 4.3890 | 0.0371* |
| 1 | -50 | -9 | 0 | 4.8663 | 0.0283* | 2 | 62 | -19 | 0 | 5.1757 | 0.0239* |
| 2 | -56 | -22 | 8 | 3.6117 | 0.0592 | 3 | 53 | -14 | 6 | 4.3633 | 0.0377* |
| 3 | -36 | -24 | 10 | 1.7009 | 0.2237 | 4 | 39 | -19 | 5 | 4.2775 | 0.0396* |
| 4 | -38 | -9 | 13 | 1.1872 | 0.3385 | 5 | 37 | -8 | 14 | 3.8743 | 0.0503 |
| 5 | -36 | -26 | 19 | 0.7475 | 0.4944 | 6 | 35 | -21 | 14 | 1.9699 | 0.1820 |
| 6 | -59 | -37 | 16 | 4.0055 | 0.0465* | 7 | 60 | -24 | 11 | 3.8020 | 0.0526 |
| 7 | -41 | -35 | 14 | 0.9520 | 0.4133 | 8 | 65 | -34 | 11 | 4.9572 | 0.0270* |
| 8 | -49 | -13 | 14 | 1.9646 | 0.1828 | 9 | 41 | -13 | 18 | 3.5853 | 0.0602 |
| 9 | -60 | -2 | 11 | 2.3017 | 0.1425 | 10 | 41 | -29 | 18 | 2.4031 | 0.1325 |
| 10 | -48 | -24 | 18 | 3.5218 | 0.0626 | 11 | 50 | -10 | 13 | 2.1099 | 0.1640 |
| 11 | -61 | -18 | 19 | 2.8074 | 0.1000 | 12 | 59 | 1 | 10 | 2.9361 | 0.0916 |
| 12 | -59 | -2 | 23 | 1.7752 | 0.2112 | 13 | 49 | -21 | 19 | 3.2509 | 0.0744 |
| 13 | -63 | -10 | 31 | 3.1213 | 0.0810 | 14 | 62 | -12 | 15 | 2.7862 | 0.1014 |
| 14 | -53 | -9 | 30 | 2.0084 | 0.1769 | 15 | 61 | 6 | 30 | 0.2075 | 0.8155 |
| 15 | -55 | -20 | 41 | 3.4734 | 0.0645 | 16 | 60 | -5 | 27 | 1.3728 | 0.2905 |
| 16 | -51 | -7 | 43 | 0.6190 | 0.5548 | 17 | 61 | -14 | 30 | 2.5794 | 0.1170 |
| 17 | -48 | -16 | 40 | 0.5773 | 0.5762 | 18 | 52 | -6 | 37 | 0.8484 | 0.4523 |
| 18 | -8 | -15 | 47 | 1.5857 | 0.2449 | 19 | 54 | -17 | 40 | 1.9797 | 0.1807 |
| 19 | -41 | -14 | 47 | 0.1235 | 0.8850 | 20 | 11 | -17 | 41 | 2.3133 | 0.1413 |
| 20 | -49 | -17 | 54 | 2.0508 | 0.1713 | 21 | 52 | -13 | 49 | 0.8666 | 0.4451 |
| 21 | -48 | -29 | 58 | 3.4623 | 0.0650 | 22 | 44 | -10 | 49 | 0.6747 | 0.5276 |
| 22 | -39 | -25 | 53 | 1.8018 | 0.2069 | 23 | 49 | -26 | 56 | 3.4618 | 0.0650 |
| 23 | -9 | -38 | 54 | 2.1369 | 0.1607 | 24 | 7 | -10 | 51 | 0.9312 | 0.4208 |
| 24 | -4 | -25 | 56 | 1.6783 | 0.2277 | 25 | 43 | -21 | 54 | 2.0080 | 0.1769 |
| 25 | -4 | -9 | 59 | 2.0786 | 0.1678 | 26 | 37 | -20 | 64 | 1.9546 | 0.1842 |
| 26 | -36 | -19 | 65 | 0.4890 | 0.6250 | 27 | 32 | -34 | 63 | 2.0160 | 0.1759 |
| 27 | -30 | -46 | 63 | 0.5356 | 0.5987 | 28 | 31 | -41 | 64 | 1.2644 | 0.3175 |
| 28 | -32 | -29 | 63 | 1.4306 | 0.2772 | 29 | 34 | -27 | 61 | 3.0721 | 0.0837 |
| 29 | -30 | -38 | 65 | 0.3446 | 0.7153 | 30 | 4 | -25 | 58 | 0.6121 | 0.5583 |
| 30 | -23 | -11 | 65 | 0.0405 | 0.9604 | 31 | 29 | -11 | 65 | 0.2222 | 0.8040 |
| 31 | -19 | -24 | 67 | 0.4583 | 0.6430 | 32 | 9 | -40 | 68 | 1.4847 | 0.2654 |
| 32 | -9 | -42 | 70 | 1.7638 | 0.2130 | 33 | 21 | -24 | 67 | 0.8626 | 0.4467 |
| 33 | -4 | -25 | 69 | 1.7332 | 0.2182 | 34 | 22 | -29 | 68 | 2.0686 | 0.1691 |
| 34 | -14 | -11 | 73 | 2.0821 | 0.1674 | 35 | 22 | -35 | 71 | 1.4674 | 0.2691 |
| 35 | -19 | -31 | 69 | 1.7024 | 0.2234 | 36 | 17 | -6 | 69 | 1.2098 | 0.3322 |
| 36 | -19 | -40 | 72 | 1.9076 | 0.1908 | 37 | 16 | -47 | 74 | 0.7629 | 0.4876 |
| 37 | -12 | -27 | 73 | 0.2907 | 0.7529 | 38 | 5 | -22 | 72 | 1.6794 | 0.2275 |
| **DorsAttn** |  |  |  |  |  | 39 | 17 | -18 | 73 | 1.4493 | 0.2730 |
| Post_1 | -45 | -42 | -21 | 0.1962 | 0.8244 | 40 | 13 | -33 | 76 | 0.8235 | 0.4622 |
| Post_2 | -49 | -56 | -15 | 1.3841 | 0.2878 | **DorsAttn** |  |  |  |  |  |
| Post_3 | -55 | -62 | -1 | 4.0065 | 0.0465* | Post_1 | 50 | -49 | -18 | 4.0450 | 0.0454* |
| Post_4 | -48 | -65 | 15 | 3.2102 | 0.0764 | Post_2 | 59 | -55 | -2 | 5.3175 | 0.0222* |
| Post_5 | -26 | -70 | 31 | 2.4349 | 0.1295 | Post_3 | 54 | -56 | 12 | 7.5412 | 0.0076* |
| Post_6 | -55 | -32 | 45 | 3.2080 | 0.0765 | Post_4 | 45 | -75 | 31 | 6.7841 | 0.0107* |
| Post_7 | -23 | -65 | 46 | 0.5577 | 0.5867 | Post_5 | 32 | -66 | 35 | 4.3905 | 0.0371* |
| Post_8 | -46 | -29 | 44 | 1.2162 | 0.3304 | Post_6 | 57 | -23 | 44 | 2.2997 | 0.1427 |
| Post_9 | -33 | -46 | 41 | 0.6271 | 0.5507 | Post_7 | 19 | -79 | 50 | 1.5203 | 0.2579 |
| Post_10 | -29 | -58 | 50 | 2.3609 | 0.1366 | Post_8 | 44 | -37 | 50 | 6.0616 | 0.0152* |
| Post_11 | -39 | -37 | 49 | 1.3096 | 0.3059 | Post_9 | 45 | -28 | 42 | 3.0541 | 0.0847 |
| Post_12 | -36 | -52 | 56 | 2.0852 | 0.1670 | Post_10 | 31 | -64 | 53 | 4.2354 | 0.0406* |
| Post_13 | -15 | -71 | 57 | 1.6499 | 0.2328 | Post_11 | 36 | -44 | 45 | 5.8551 | 0.0168* |
| Post_14 | -29 | -61 | 62 | 1.0329 | 0.3856 | Post_12 | 21 | -69 | 53 | 1.4206 | 0.2794 |
| Post_15 | -7 | -59 | 63 | 0.8774 | 0.4409 | Post_13 | 35 | -36 | 51 | 1.2533 | 0.3204 |
| Post_16 | -20 | -57 | 66 | 0.9727 | 0.4060 | Post_14 | 34 | -50 | 54 | 4.0553 | 0.0451* |
| Post_17 | -13 | -50 | 72 | 0.1191 | 0.8888 | Post_15 | 8 | -71 | 53 | 5.5840 | 0.0193* |
| FEF_1 | -40 | -3 | 51 | 1.1532 | 0.3483 | Post_16 | 27 | -58 | 61 | 1.5573 | 0.2504 |
| FEF_2 | -25 | -1 | 55 | 0.9209 | 0.4246 | Post_17 | 7 | -54 | 59 | 2.5993 | 0.1154 |
| FEF_3 | -30 | -8 | 52 | 0.0255 | 0.9749 | Post_18 | 14 | -64 | 65 | 1.4645 | 0.2697 |
| FEF_4 | -21 | 5 | 65 | 1.9622 | 0.1831 | Post_19 | 24 | -50 | 68 | 1.2121 | 0.3315 |
| PrCv_1 | -49 | 6 | 26 | 2.1728 | 0.1566 | FEF_1 | 39 | -3 | 53 | 0.7786 | 0.4809 |
| PrCv_2 | -50 | 3 | 38 | 0.6354 | 0.5466 | FEF_2 | 27 | -3 | 52 | 0.6605 | 0.5344 |
| **SalVentAttn** |  |  |  |  |  | FEF_3 | 25 | -3 | 64 | 0.9091 | 0.4289 |
| ParOper_1 | -55 | -32 | 22 | 4.0199 | 0.0461* | PrCv_1 | 49 | 8 | 25 | 1.8681 | 0.1966 |
| ParOper_2 | -58 | -44 | 27 | 5.1187 | 0.0247* | **SalVentAttn** |  |  |  |  |  |
| ParOper_3 | -61 | -23 | 33 | 4.3142 | 0.0388* | TempOccPar_1 | 59 | -46 | 7 | 5.8933 | 0.0165* |
| ParOper_4 | -61 | -36 | 33 | 7.0576 | 0.0094* | TempOccPar_2 | 51 | -41 | 13 | 4.3501 | 0.0380* |
| TempOcc_1 | -57 | -54 | 10 | 4.6227 | 0.0325* | TempOccPar_3 | 62 | -40 | 22 | 3.1288 | 0.0806 |
| FrOperIns_1 | -44 | 5 | -17 | 1.0301 | 0.3865 | TempOccPar_4 | 58 | -31 | 24 | 3.9993 | 0.0467* |
| FrOperIns_2 | -39 | 2 | -4 | 2.6000 | 0.1153 | TempOccPar_5 | 60 | -22 | 22 | 4.1303 | 0.0432* |
| FrOperIns_3 | -33 | 25 | -1 | 1.3863 | 0.2873 | TempOccPar_6 | 63 | -26 | 38 | 3.2698 | 0.0735 |
| FrOperIns_4 | -40 | -15 | -2 | 3.1954 | 0.0772 | TempOccPar_7 | 62 | -37 | 37 | 3.4765 | 0.0644 |
| FrOperIns_5 | -33 | 19 | 8 | 2.3695 | 0.1357 | PrC_1 | 51 | 3 | 41 | 0.1071 | 0.8992 |
| FrOperIns_6 | -36 | 4 | 11 | 0.9007 | 0.4321 | FrOperIns_1 | 40 | 5 | -15 | 1.5950 | 0.2431 |
| FrOperIns_7 | -43 | 12 | 2 | 1.7598 | 0.2137 | FrOperIns_2 | 41 | 8 | -3 | 4.1891 | 0.0417* |
| FrOperIns_8 | -50 | 1 | 5 | 1.5681 | 0.2483 | FrOperIns_3 | 40 | -10 | -4 | 5.4433 | 0.0208* |
| FrOperIns_9 | -52 | 9 | 13 | 1.7536 | 0.2147 | FrOperIns_4 | 39 | -2 | 6 | 4.8220 | 0.0290* |
| PFCl_1 | -29 | 43 | 30 | 1.5913 | 0.2438 | FrOperIns_5 | 37 | 23 | 5 | 7.2081 | 0.0088* |
| Med_1 | -6 | 22 | 31 | 3.9710 | 0.0475* | FrOperIns_6 | 38 | 7 | 11 | 4.6448 | 0.0321* |
| Med_2 | -7 | 0 | 41 | 1.4131 | 0.2811 | FrOperIns_7 | 49 | 5 | 3 | 3.6947 | 0.0562 |
| Med_3 | -11 | -27 | 41 | 2.1605 | 0.1580 | FrOperIns_8 | 54 | 12 | 12 | 4.1237 | 0.0433* |
| Med_4 | -5 | 9 | 48 | 2.0092 | 0.1768 | PFCl_1 | 33 | 45 | 28 | 9.0032 | 0.0041* |
| Med_5 | -13 | -41 | 47 | 2.6658 | 0.1102 | Med_1 | 7 | 19 | 35 | 3.8508 | 0.0511 |
| Med_6 | -6 | -49 | 57 | 3.1599 | 0.0790 | Med_2 | 7 | 2 | 43 | 1.3557 | 0.2946 |
| Med_7 | -8 | -3 | 71 | 3.9149 | 0.0491* | Med_3 | 12 | -34 | 43 | 3.0737 | 0.0836 |
| **Limbic** |  |  |  |  |  | Med_4 | 6 | 11 | 58 | 1.8443 | 0.2003 |
| OFC_1 | -12 | 25 | -21 | 0.7001 | 0.5157 | Med_5 | 10 | -43 | 53 | 1.5161 | 0.2588 |
| OFC_2 | -24 | 23 | -20 | 1.1197 | 0.3582 | Med_6 | 11 | -32 | 50 | 1.5528 | 0.2513 |
| OFC_3 | -10 | 47 | -21 | 0.4392 | 0.6545 | Med_7 | 7 | -2 | 67 | 2.5121 | 0.1227 |
| OFC_4 | -4 | 23 | -19 | 2.1224 | 0.1625 | Med_8 | 16 | 7 | 69 | 0.0601 | 0.9419 |
| OFC_5 | -15 | 65 | -8 | 1.6398 | 0.2346 | **Limbic** |  |  |  |  |  |
| TempPole_1 | -37 | -5 | -42 | 0.8429 | 0.4544 | OFC_1 | 13 | 24 | -21 | 5.5839 | 0.0193* |
| TempPole_2 | -25 | 6 | -39 | 0.5995 | 0.5647 | OFC_2 | 23 | 22 | -21 | 8.2552 | 0.0056* |
| TempPole_3 | -26 | -9 | -33 | 0.0388 | 0.9621 | OFC_3 | 8 | 47 | -23 | 2.9997 | 0.0878 |
| TempPole_4 | -54 | -21 | -31 | 3.1923 | 0.0773 | OFC_4 | 20 | 43 | -18 | 1.7298 | 0.2187 |
| TempPole_5 | -40 | -21 | -27 | 0.1080 | 0.8985 | OFC_5 | 5 | 22 | -21 | 3.7530 | 0.0542 |
| TempPole_6 | -44 | 13 | -34 | 0.4082 | 0.6737 | OFC_6 | 9 | 63 | -14 | 5.6390 | 0.0188* |
| TempPole_7 | -32 | 12 | -29 | 0.1963 | 0.8244 | TempPole_1 | 28 | -1 | -40 | 0.3657 | 0.7012 |
| TempPole_8 | -21 | -21 | -26 | 0.0375 | 0.9633 | TempPole_2 | 49 | -7 | -39 | 3.2085 | 0.0765 |
| **Cont** |  |  |  |  |  | TempPole_3 | 37 | 17 | -38 | 0.0830 | 0.9209 |
| Par_1 | -29 | -74 | 42 | 2.6477 | 0.1116 | TempPole_4 | 39 | -15 | -31 | 0.4962 | 0.6208 |
| Par_2 | -58 | -42 | 45 | 8.4326 | 0.0052* | TempPole_5 | 29 | 12 | -30 | 1.2562 | 0.3196 |
| Par_3 | -53 | -50 | 45 | 3.5244 | 0.0625 | TempPole_6 | 50 | -28 | -26 | 5.7944 | 0.0173* |
| Par_4 | -35 | -62 | 48 | 1.1535 | 0.3482 | TempPole_7 | 23 | -18 | -27 | 0.0936 | 0.9113 |
| Par_5 | -42 | -52 | 49 | 2.9130 | 0.0931 | **Cont** |  |  |  |  |  |
| Par_6 | -45 | -41 | 47 | 5.8417 | 0.0169* | Par_1 | 54 | -53 | 44 | 4.6626 | 0.0317* |
| Temp_1 | -60 | -49 | -10 | 1.3700 | 0.2912 | Par_2 | 56 | -41 | 48 | 2.6146 | 0.1142 |
| OFC_1 | -27 | 49 | -14 | 1.3613 | 0.2932 | Par_3 | 35 | -71 | 47 | 4.3674 | 0.0376* |
| PFCl_1 | -42 | 49 | -6 | 0.6813 | 0.5245 | Par_4 | 54 | -33 | 51 | 1.1768 | 0.3414 |
| PFCl_2 | -48 | 35 | 10 | 2.8928 | 0.0943 | Par_5 | 47 | -44 | 46 | 1.9697 | 0.1821 |
| PFCl_3 | -38 | 49 | 11 | 2.7415 | 0.1046 | Par_6 | 41 | -55 | 48 | 5.4909 | 0.0203* |
| PFCl_4 | -28 | 57 | 13 | 2.1273 | 0.1619 | Temp_1 | 62 | -28 | -20 | 3.9542 | 0.0480* |
| PFCl_5 | -42 | 38 | 22 | 3.9778 | 0.0473* | Temp_2 | 63 | -42 | -11 | 2.4229 | 0.1307 |
| PFCl_6 | -45 | 20 | 27 | 1.1300 | 0.3551 | PFCv_1 | 34 | 21 | -8 | 2.4088 | 0.1320 |
| PFCl_7 | -39 | 7 | 34 | 1.8778 | 0.1952 | PFCl_1 | 28 | 55 | -14 | 2.1334 | 0.1612 |
| PFCl_8 | -36 | 32 | 38 | 4.6774 | 0.0315* | PFCl_2 | 42 | 51 | -6 | 2.2059 | 0.1528 |
| PFCv_1 | -34 | 16 | -8 | 2.0773 | 0.1680 | PFCl_3 | 27 | 59 | 3 | 6.4347 | 0.0126* |
| pCun_1 | -9 | -77 | 45 | 3.4833 | 0.0641 | PFCl_4 | 49 | 40 | 5 | 2.5706 | 0.1177 |
| pCun_2 | -5 | -64 | 52 | 4.3561 | 0.0378* | PFCl_5 | 42 | 46 | 14 | 6.7489 | 0.0109* |
| Cing_1 | -3 | 5 | 29 | 2.7796 | 0.1019 | PFCl_6 | 50 | 30 | 18 | 1.8824 | 0.1945 |
| Cing_2 | -4 | -22 | 29 | 1.8890 | 0.1935 | PFCl_7 | 48 | 18 | 23 | 2.0080 | 0.1769 |
| PFCmp_1 | -4 | 28 | 47 | 1.5546 | 0.2510 | PFCl_8 | 25 | 54 | 25 | 5.1106 | 0.0248* |
| **Default** |  |  |  |  |  | PFCl_9 | 47 | 29 | 28 | 1.9672 | 0.1824 |
| Temp_1 | -54 | -2 | -30 | 1.0735 | 0.3725 | PFCl_10 | 39 | 11 | 34 | 4.5877 | 0.0331* |
| Temp_2 | -62 | -18 | -21 | 1.4833 | 0.2657 | PFCl_11 | 39 | 33 | 38 | 4.8113 | 0.0292* |
| Temp_3 | -60 | -36 | -18 | 3.0520 | 0.0848 | PFCl_12 | 45 | 19 | 44 | 2.1639 | 0.1576 |
| Temp_4 | -57 | -9 | -14 | 1.5111 | 0.2598 | PFCl_13 | 43 | 7 | 51 | 2.5487 | 0.1195 |
| Temp_5 | -53 | 6 | -11 | 1.9090 | 0.1906 | PFCl_14 | 34 | 15 | 56 | 2.0459 | 0.1720 |
| Temp_6 | -61 | -35 | -3 | 2.7368 | 0.1049 | PFCl_15 | 24 | 10 | 58 | 6.1502 | 0.0145* |
| Temp_7 | -52 | -22 | -6 | 1.4847 | 0.2654 | pCun_1 | 13 | -71 | 39 | 3.7635 | 0.0539 |
| Temp_8 | -61 | -13 | -3 | 3.8946 | 0.0497* | pCun_2 | 5 | -64 | 44 | 6.5491 | 0.0119* |
| Temp_9 | -62 | -32 | 5 | 1.9955 | 0.1786 | Cing_1 | 6 | -26 | 28 | 3.0411 | 0.0854 |
| Temp_10 | -52 | -43 | 5 | 1.9554 | 0.1841 | Cing_2 | 5 | 1 | 30 | 2.3282 | 0.1398 |
| Par_1 | -59 | -49 | 16 | 3.1610 | 0.0789 | PFCmp_1 | 8 | 35 | 25 | 2.3776 | 0.1350 |
| Par_2 | -45 | -58 | 21 | 3.4381 | 0.0660 | PFCmp_2 | 5 | 28 | 48 | 0.6212 | 0.5537 |
| Par_3 | -40 | -79 | 30 | 3.0626 | 0.0842 | **Default** |  |  |  |  |  |
| Par_4 | -47 | -64 | 31 | 3.3261 | 0.0709 | Par_1 | 55 | -46 | 19 | 6.6472 | 0.0114* |
| Par_5 | -57 | -55 | 30 | 4.1434 | 0.0428* | Par_2 | 48 | -64 | 22 | 3.8054 | 0.0525 |
| Par_6 | -41 | -72 | 43 | 1.6782 | 0.2277 | Par_3 | 53 | -53 | 26 | 4.8096 | 0.0292* |
| Par_7 | -49 | -60 | 47 | 1.7563 | 0.2143 | Par_4 | 55 | -45 | 33 | 7.8287 | 0.0067* |
| PFC_1 | -36 | 22 | -16 | 0.9194 | 0.4251 | Par_5 | 47 | -64 | 42 | 2.3604 | 0.1366 |
| PFC_2 | -36 | 37 | -13 | 0.6093 | 0.5597 | Temp_1 | 49 | 9 | -33 | 1.2784 | 0.3138 |
| PFC_3 | -5 | 55 | -10 | 1.0803 | 0.3703 | Temp_2 | 61 | -8 | -23 | 4.6755 | 0.0315* |
| PFC_4 | -6 | 35 | -9 | 2.8208 | 0.0990 | Temp_3 | 47 | 16 | -20 | 1.1880 | 0.3383 |
| PFC_5 | -46 | 32 | -10 | 0.5829 | 0.5733 | Temp_4 | 55 | -4 | -14 | 6.3583 | 0.0131* |
| PFC_6 | -28 | 58 | -1 | 2.4262 | 0.1304 | Temp_5 | 63 | -23 | -7 | 4.8062 | 0.0293* |
| PFC_7 | -48 | 28 | 0 | 0.3276 | 0.7269 | Temp_6 | 49 | -20 | -8 | 2.7638 | 0.1030 |
| PFC_8 | -6 | 59 | 7 | 3.8473 | 0.0512 | Temp_7 | 63 | -38 | 0 | 3.8078 | 0.0524 |
| PFC_9 | -6 | 45 | 6 | 2.3660 | 0.1361 | Temp_8 | 50 | -33 | 2 | 2.4384 | 0.1292 |
| PFC_10 | -53 | 19 | 11 | 1.3044 | 0.3072 | PFCv_1 | 35 | 23 | -18 | 1.1536 | 0.3481 |
| PFC_11 | -16 | 67 | 8 | 1.9139 | 0.1899 | PFCv_2 | 35 | 38 | -13 | 3.1150 | 0.0814 |
| PFC_12 | -5 | 34 | 21 | 2.6529 | 0.1112 | PFCv_3 | 48 | 32 | -8 | 2.0389 | 0.1729 |
| PFC_13 | -4 | 51 | 28 | 2.5868 | 0.1164 | PFCv_4 | 54 | 24 | 6 | 1.9971 | 0.1784 |
| PFC_14 | -14 | 58 | 31 | 1.0616 | 0.3763 | PFCdPFCm_1 | 5 | 41 | -11 | 5.8971 | 0.0165* |
| PFC_15 | -22 | 51 | 31 | 0.8613 | 0.4472 | PFCdPFCm_2 | 9 | 67 | 1 | 1.4291 | 0.2775 |
| PFC_16 | -25 | 28 | 43 | 1.4786 | 0.2667 | PFCdPFCm_3 | 7 | 42 | 4 | 3.5758 | 0.0605 |
| PFC_17 | -41 | 19 | 48 | 1.8250 | 0.2032 | PFCdPFCm_4 | 7 | 54 | 13 | 4.0193 | 0.0461* |
| PFC_18 | -8 | 43 | 51 | 0.2417 | 0.7890 | PFCdPFCm_5 | 17 | 65 | 16 | 4.1255 | 0.0433* |
| PFC_19 | -18 | 36 | 48 | 0.8108 | 0.4674 | PFCdPFCm_6 | 6 | 25 | 18 | 2.5392 | 0.1203 |
| PFC_20 | -42 | 7 | 48 | 1.9570 | 0.1838 | PFCdPFCm_7 | 6 | 58 | 29 | 2.5830 | 0.1167 |
| PFC_21 | -30 | 14 | 57 | 2.0953 | 0.1658 | PFCdPFCm_8 | 16 | 52 | 36 | 2.7233 | 0.1059 |
| PFC_22 | -22 | 20 | 52 | 2.0594 | 0.1703 | PFCdPFCm_9 | 5 | 44 | 40 | 1.0090 | 0.3935 |
| PFC_23 | -13 | 24 | 61 | 1.1463 | 0.3503 | PFCdPFCm_10 | 26 | 34 | 39 | 2.8906 | 0.0945 |
| PFC_24 | -6 | 10 | 65 | 1.5514 | 0.2516 | PFCdPFCm_11 | 14 | 39 | 52 | 0.7203 | 0.5065 |
| pCunPCC_1 | -8 | -52 | 9 | 1.6798 | 0.2274 | PFCdPFCm_12 | 24 | 26 | 51 | 3.5612 | 0.0611 |
| pCunPCC_2 | -13 | -61 | 19 | 1.3833 | 0.2880 | PFCdPFCm_13 | 12 | 20 | 63 | 1.0441 | 0.3819 |
| pCunPCC_3 | -4 | -53 | 20 | 2.0058 | 0.1772 | pCunPCC_1 | 12 | -55 | 15 | 2.5119 | 0.1227 |
| pCunPCC_4 | -6 | -41 | 24 | 3.8292 | 0.0517 | pCunPCC_2 | 7 | -44 | 20 | 2.6971 | 0.1078 |
| pCunPCC_5 | -10 | -70 | 32 | 1.8996 | 0.1920 | pCunPCC_3 | 17 | -63 | 28 | 2.7437 | 0.1044 |
| pCunPCC_6 | -5 | -60 | 30 | 2.4130 | 0.1316 | pCunPCC_4 | 6 | -52 | 23 | 3.4659 | 0.0649 |
| pCunPCC_7 | -7 | -44 | 32 | 3.4972 | 0.0636 | pCunPCC_5 | 5 | -63 | 31 | 3.7418 | 0.0546 |
| pCunPCC_8 | -4 | -34 | 38 | 3.7272 | 0.0551 | pCunPCC_6 | 7 | -39 | 35 | 3.0256 | 0.0863 |
| pCunPCC_9 | -3 | -15 | 37 | 3.5660 | 0.0609 | pCunPCC_7 | 4 | -20 | 37 | 4.3340 | 0.0383* |
| pCunPCC_10 | -3 | -68 | 41 | 3.2322 | 0.0754 | pCunPCC_8 | 10 | -53 | 35 | 4.2878 | 0.0394* |

Note: * indicates p < 0.05 (uncorrected for multiple comparisons). Vis, Visual network; SomMot, Somatomotor network; DorsAttn, Dorsal Attention network; SalVentAttn, Salience/Ventral Attention network; Limbic, Limbic network; Cont, Frontoparietal Control network; Default, Default Mode network. LH and RH indicate the left and right hemispheres, respectively.
