# Supplementary material for: Effects of Kuanxiong aerosol for preventing acute mountain sickness: an exploratory small-sample randomized, placebo-controlled trial
Source: Front Physiol. 2026 Jul 21;17:1834301. doi: 10.3389/fphys.2026.1834301 (PMC13433183; doi:10.3389/fphys.2026.1834301)
Supplement: Supplementary file 4 [file Table4.docx]

**Supplementary Table 4. Post hoc ROI‑level comparisons of CBF between sessions in the exploratory voluntary additional session (self‑controlled)**

| **Hypobaric hypoxic exposure without KXA vs. Baseline** | | | | | **Hypobaric hypoxic exposure with KXA vs. Baseline** | | | | |
| --- | --- | --- | --- | --- | --- | --- | --- | --- | --- |
| **ROI name** | **Hemi** | **Network** | ***T* value** | ***P* value** | **ROI name** | **Hemi** | **Network** | ***T* value** | ***p* value** |
| PFCv_1 | LH | Cont | 4.3099 | 0.0050 | 31 | LH | Vis | 4.1025 | 0.0063 |
| 17 | RH | Vis | 3.3528 | 0.0154 | 1 | LH | SomMot | 3.5945 | 0.0114 |
| Post_1 | RH | DorsAttn | 3.3786 | 0.0149 | 7 | LH | SomMot | 3.4745 | 0.0132 |
| Post_13 | RH | DorsAttn | 5.3352 | 0.0018 | 8 | LH | SomMot | 4.6140 | 0.0036 |
| Post_14 | RH | DorsAttn | 3.4109 | 0.0143 | 9 | LH | SomMot | 3.8678 | 0.0083 |
| FrOperIns_2 | RH | SalVentAttn | 3.3831 | 0.0148 | 11 | LH | SomMot | 3.5411 | 0.0122 |
| OFC_1 | RH | Limbic | 3.8917 | 0.0081 | 12 | LH | SomMot | 6.1620 | 0.0008 |
| PFCl_2 | RH | Cont | 4.0417 | 0.0068 | 14 | LH | SomMot | 3.5800 | 0.0116 |
| PFCl_10 | RH | Cont | 3.5668 | 0.0118 | ParOper_2 | LH | SalVentAttn | 5.1549 | 0.0021 |
| Temp_1 | RH | Default | 3.7981 | 0.0090 | ParOper_3 | LH | SalVentAttn | 5.2759 | 0.0019 |
| Temp_3 | RH | Default | 3.3254 | 0.0159 | FrOperIns_3 | LH | SalVentAttn | 5.0376 | 0.0024 |
|  |  |  |  |  | Par_1 | LH | Cont | 3.7046 | 0.0100 |
|  |  |  |  |  | PFCl_4 | LH | Cont | 3.5314 | 0.0123 |
|  |  |  |  |  | PFCl_7 | LH | Cont | 3.5967 | 0.0114 |
|  |  |  |  |  | 28 | RH | SomMot | 7.5491 | 0.0003 |
|  |  |  |  |  | 33 | RH | SomMot | 3.3914 | 0.0147 |
|  |  |  |  |  | Post_2 | RH | DorsAttn | 3.5801 | 0.0116 |
|  |  |  |  |  | FrOperIns_4 | RH | SalVentAttn | 3.4595 | 0.0135 |
|  |  |  |  |  | Med_6 | RH | SalVentAttn | 3.5075 | 0.0127 |

Note: All P values were not corrected at the ROI level; however, a family-wise error (FWE) correction was applied across multiple session comparisons, with a threshold of *p* < 0.05/3. Only ROIs that survived this session-wise correction are presented. Vis, Visual network; SomMot, Somatomotor network; DorsAttn, Dorsal Attention network; SalVentAttn, Salience/Ventral Attention network; Limbic, Limbic network; Cont, Frontoparietal Control network; Default, Default Mode network. LH and RH indicate the left and right hemispheres, respectively.
